# Whole genome sequencing-based association study to unravel genetic architecture of cooked grain width and length traits in rice

**DOI:** 10.1038/s41598-017-12778-6

**Published:** 2017-09-29

**Authors:** Gopal Misra, Saurabh Badoni, Roslen Anacleto, Andreas Graner, Nickolai Alexandrov, Nese Sreenivasulu

**Affiliations:** 1 Grain Quality and Nutrition Center, Plant Breeding Division, International Rice Research Institute, DAPO Box 7777, Metro Manila, 1301 Philippines; 2 Genetics and Biotechnology Division, International Rice Research Institute, DAPO Box 7777, Metro Manila, 1301 Philippines; 3 Leibniz institute of Plant Genetics and Crop Plant Research (IPK), Corrensstrasse 03, 06466 Gatersleben, Germany

## Abstract

In this study, we used 2.9 million single nucleotide polymorphisms (SNP) and 393,429 indels derived from whole genome sequences of 591 rice landraces to determine the genetic basis of cooked and raw grain length, width and shape using genome-wide association study (GWAS). We identified a unique fine-mapped genetic region GWi7.1 significantly associated with cooked and raw grain width. Additionally, GWi7.2 that harbors *GL7*/*GW7* a cloned gene for grain dimension was found. Novel regions in chromosomes 10 and 11 were also found to be associated with cooked grain shape and raw grain width, respectively. The indel-based GWAS identified fine-mapped genetic regions GL3.1 and GWi5.1 that matched synteny breakpoints between *indica* and *japonica*. GL3.1 was positioned a few kilobases away from *GS*3, a cloned gene for cooked and raw grain lengths in *indica*. GWi5.1 found to be significantly associated with cooked and raw grain width. It anchors upstream of cloned gene *GW*5, which varied between *indica* and *japonica* accessions. GWi11.1 is present inside the 3′-UTR of a functional gene in *indica* that corresponds to a syntenic break in chromosome 11 of *japonica*. Our results identified novel allelic structural variants and haplotypes confirmed using single locus and multilocus SNP and indel-based GWAS.

## Introduction

Rice (*Oryza sativa* L.) being the most important food crop for more than half of the world’s population fulfills 45–70% of the daily caloric requirement of rice consumers in Asia (http://ricepedia.org/rice-around-the-world/asia). Rice accumulated substantial genetic variation during domestication leading to differences in seed morphology. This rice domestication process that occurred in various rice-eating cultures in Asian societies has led to create substantial genetic variability between *indica* and *japonica* subspecies^1,2^. Specific allelic combinations of agronomically important genes have been selected over others that led to huge phenotypic variation for traits including grain dimensions and shape at the subspecies level^3–8^. The key grain quality traits that factor into different consumer preferences are grain length, width, and grain shape (ratio between grain length and width). Variation in *indica* and *japonica* subspecies is also evident through the specific conserved allelic variants of major genes such as *grain size 3* (*GS3*) and *grain width 5* (*GW5*)^9–11^. Based on observed inferences of grain dimensional characteristics, *indica* rice must have undergone positive selection for long grains while *japonica* for relatively shorter and bold grains^12^. In the case of *tropical japonica* (also termed as *javanica* rice), they have larger grains than that of *temperate japonica* varieties^13,14^. The genetic basis of huge variations for grain dimensions and shape at the subspecies level arose from chromosomal rearrangements and duplications resulting in altering syntenic relationship between the subspecies^5,6,8,15^.

The evolutionary and domestication histories of rice made it best suited for GWAS^7^. This technique has been extensively used in rice as an efficient strategy for the genetic analysis of complex traits that include grain dimensions^3–5,7,8,12,16^. High-resolution dissection of universal- and population-specific large effect alleles in GWAS aids in the understanding of locally adapted allele complexes in different subspecies^7^. In addition, targeted gene association study (TGAS) has emerged as a complementary strategy to harvest other allelic variations within the candidate genes underlying genomic regions that were implicated in preceding GWAS analyses. Hence, combining GWAS with an integrated approach of targeted haplotyping on candidate gene from TGAS is considered an effective strategy for the genetic dissection of complex quantitative agronomically important traits in rice^4,5,7,8,16–18^. Published GWAS on rice traits had always been on SNP-based genotyping data that practically missed out on the importance of capturing contributions of structural variations across sub-species in rice, which may be able to explain a fraction of missing heritability computed from mixed linear model SNP-based GWAS.

The grain dimensions and shape after cooking are among the major determinants of consumer preference, especially for rice-eating cultures that prefer non-bold varieties. Very limited studies have been conducted to identify the genetic causation of grain dimensions and shape in cooked rice^19–21^. Hence, this study was focused on providing novel genetic information that could help explain further the variation in cooked and raw grain length, width and shape. Complementing GWAS with TGAS using high-resolution SNP- and indel-based genotype data on 591 diverse germplasm from the 3,000 Rice Genomes^22^ was seen as a robust and reliable analytical framework for the genetic dissection of these traits. Novel allelic variants identified from fine-mapped genetic regions on chromosomes 5 and 7 formed haplotypes that explained specific phenotypic ranges for cooked and raw grain width, while that in chromosome 11 specifically for raw grain width and chromosome 10 for cooked grain shape. Synteny between *indica* and *japonica* subspecies analyzed on the genic regions implicated through GWAS and TGAS revealed rich structural variations that deepen the genetic understanding of grain shape related traits in these pervasively cultivated rice subspecies. These haplotypes and structural variants will be useful targets for breeding programs to address the shape and dimensions of cooked rice.

## Results

### Phenotypic variation of diversity panel for cooked and raw grain length, width and shape traits

A wide range of values for grain length, width and shape were observed both for raw and cooked grains in the diversity panel (Fig. S1). Unlike breeding lines^12^, the frequency distributions of the grain dimensions and shape of *indica* landraces was not only enriched primarily for grain length but depicted variability for favored bold grains as well. In *indica* accessions, cooked grain length (GL_c_) ranged from 7.0 to 14.0 mm and raw grain length (GL) ranged between 4.31 to 7.56 mm with the average value of 5.96. Cooked grain width (GWi_c_) was narrower in *indica* ranging from 2.5 to 4.25 mm, and raw grain width (GWi) was between 1.79 to 3.09 mm with the mean value of 2.43 mm (Fig. S1A,B). Among *japonica* genotypes, GL_c_ ranged from 8.0-15.0 mm and GL from 4.1 to 7.33 mm with mean 5.73 mm. The phenotype variability in *japonica* for GWi_c_ ranged from 3.0 to 4.6 mm and GWi from 1.87 to 3.31mm with the mean 2.71 mm (Fig. S1C,D). For cooked grain length-to-width ratio (grain shape, GS_c_), the means across *indica* and *japonica* accessions were 3.0 and 2.85 mm, respectively (Fig. S1). The phenotypic variations in *indica* germplasm panel were approximately normally distributed for all three traits. However, skewed phenotypic distributions were observed in GWi and GS in *japonica* subspecies so data were transformed prior to GWAS (Fig. S2A–D).

### Identification of cooked and raw grain length, width and shape associated genetic variants through SNP-based genome wide association study

**Figure 1:**
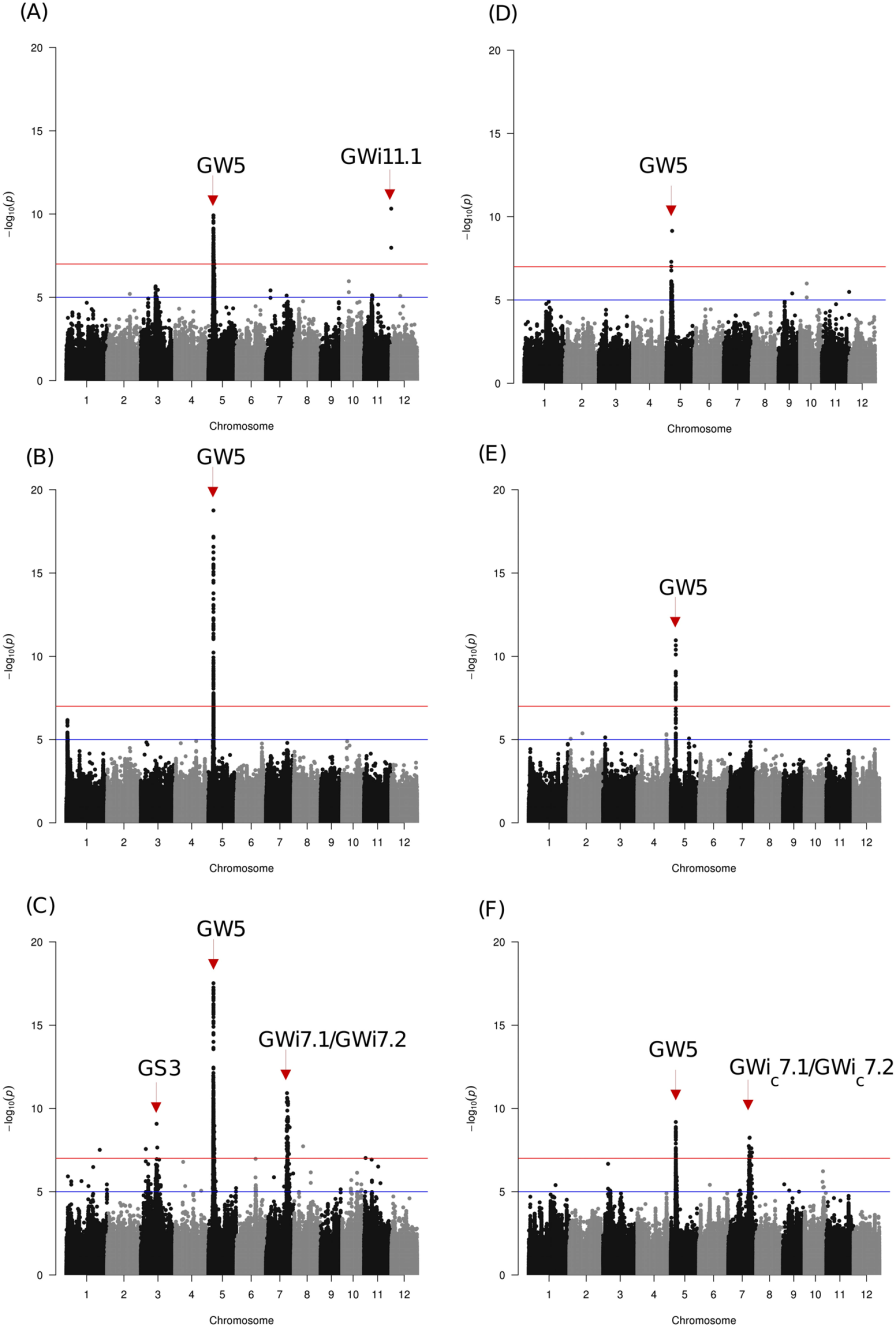
SNP-based GWAS for grain width (GWi) that confirmed *GW5* and identified prominent candidate loci on chromosomes 7 and 11. Manhattan plots of the genome-wide association studies on GWi for *japonica* (**A**), *indica* (**B**) and all (**C**, combined) panels (left side) for raw grain; *Japonica* (**D**), *indica* (**E**) and all (**F**, combined) for cooked grain (right side). The novel genomic regions in chromosomes 7 (GWi7.1, GWi7.2, GWi_c_7.1, GWi_c_7.2) and 11 (GWi11.1) were detected along with previously characterized/cloned gene in chromosome 5 (*GW5*). Horizontal red and blue line represents the genome-wide significant threshold −log_10_(*P*) value of 7 and 5, respectively.

**Figure 2:**
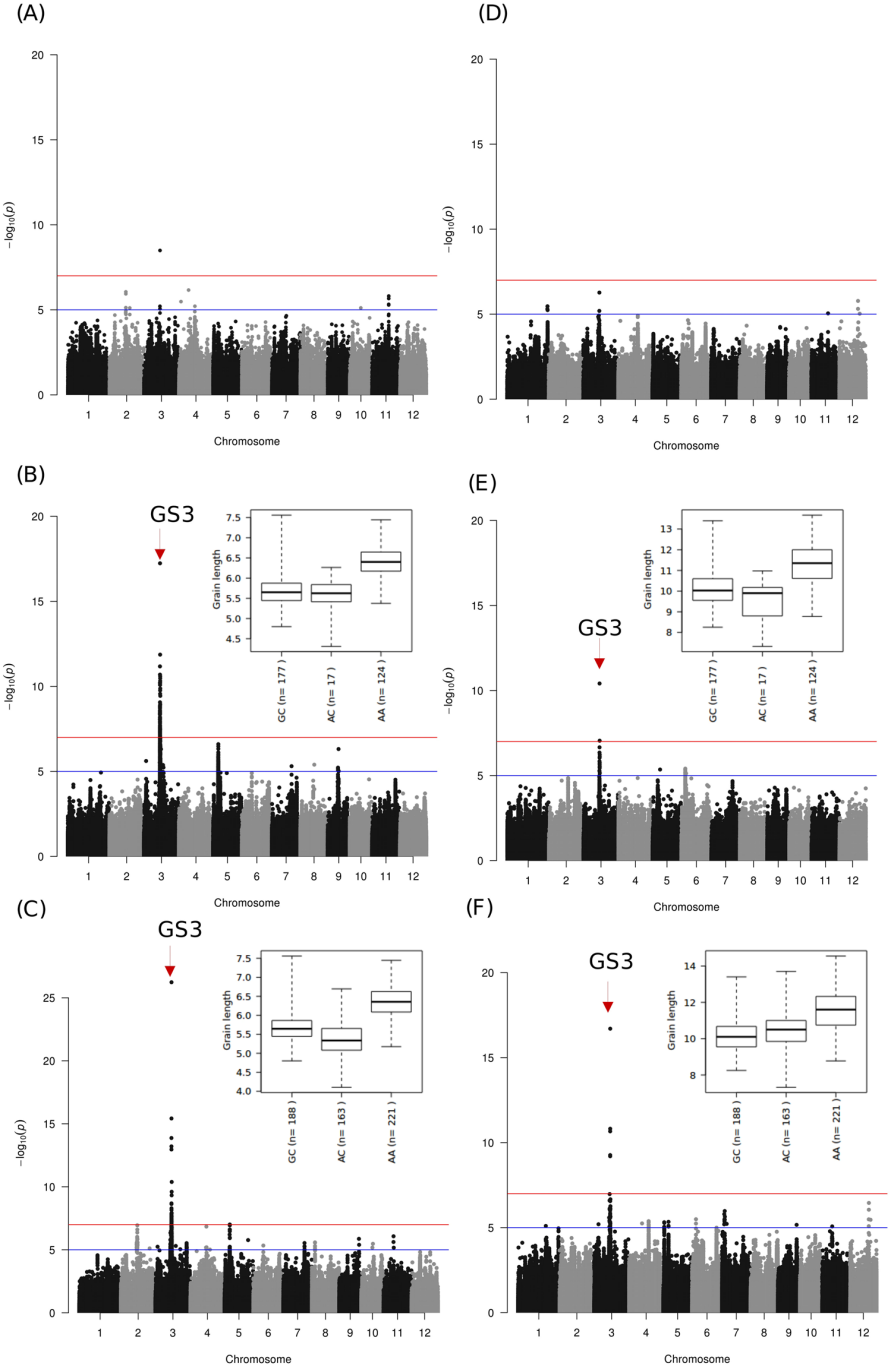
SNP-based GWAS for grain length (GL) that confirmed *GS3*. Manhattan plots of the genome-wide association studies on GL for *japonica* (**A**,**D**), *indica* (**B**,**E**) and combined (**C**,**F**) panel in raw and cooked grain, respectively. The topmost significant SNP (snp_03_16733441) in both raw and cooked grains was detected within the genic region of *GS3*. The boxplot (mentioned in **B**,**C**,**E** and **F**) visualized phenotypic variations in raw grain length (**B**,**C**) in *indica* and cooked grain length (**E**,**F**) in the combined *indica* and *japonica* panel for each haplotype formed by two of the most significant SNPs. The haplotype with the A allele of the most significant SNP (**C** to **A**) had the highest contribution to the grain length variation in both raw and cooked grains. Horizontal red and blue line represents the genome-wide significant threshold −log_10_(*P*) value of 7 and 5, respectively.

**Figure 3:**
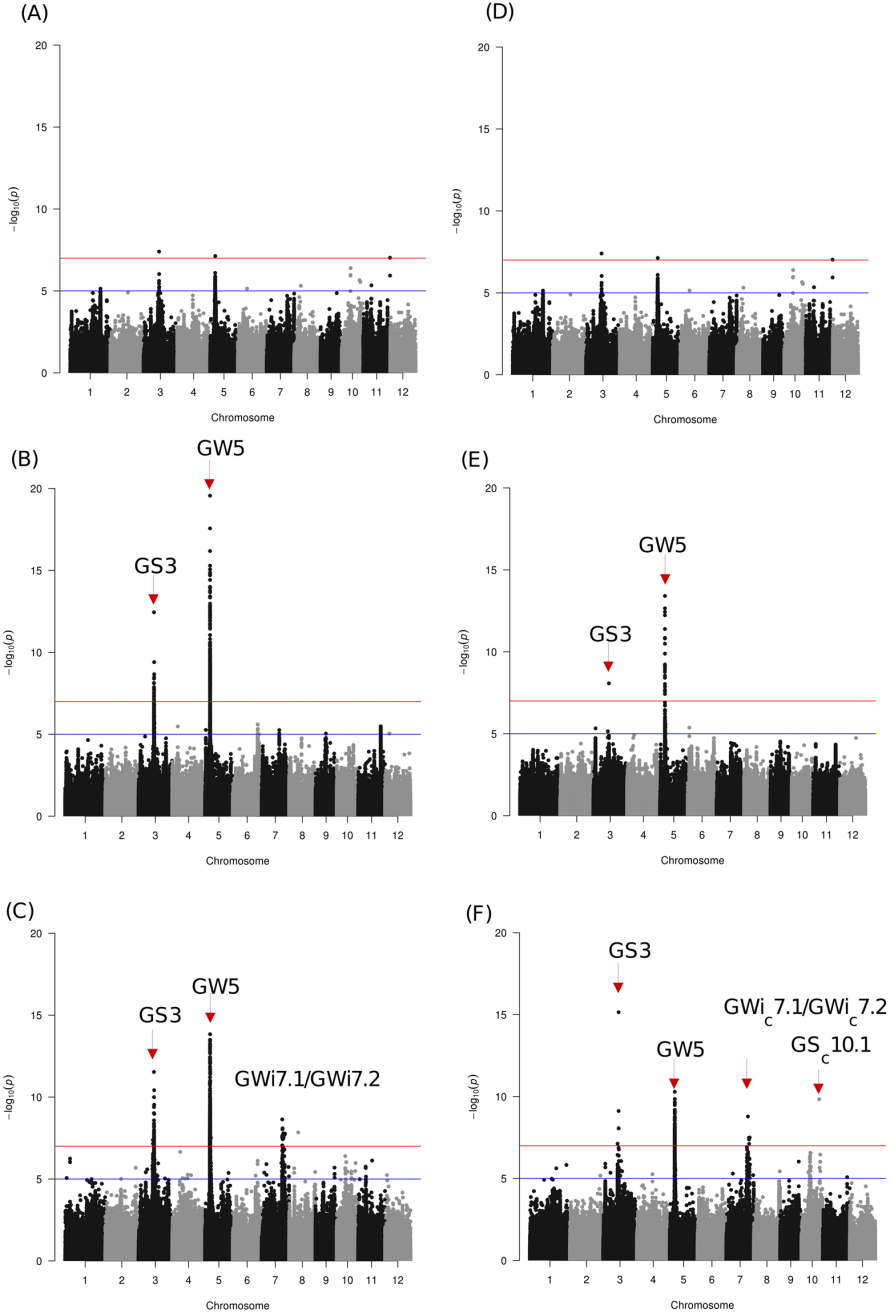
SNP-based GWAS for grain shape (GS) and association signals detected on chromosome 3, 5, 7, 10 and 11. GS being a derived trait from the ratio of GL to GWi, association signals detected in both GL and GWi were expected to show up in GS. Results showed that the cloned genes for GWi (*GW5*) and GL (*GS3*) were detected, and new candidate loci in chromosomes 3, 7 and 11 detected previously in GW and GL GWAS were also detected in *japonica* (**A**,**D**), *indica* (**B**,**E**) and all (**C**,**F** combined) panel (left side) in raw (left) and cooked grain (right), respectively. A new locus in chromosome 10 (GS_c_10.1) was also detected. Horizontal red and blue line represents the genome-wide significant threshold −log_10_(*P*) value of 7 and 5, respectively.

The high-quality re-sequencing data generated from 591 gene bank accessions composed of *indica*, *temperate* and *tropical japonica* subspecies from 72 countries^22^ representing global genetic diversity were used in this study. SNPs were called using the *japonica* (Nipponbare release 7) reference genome. Genetic structure and linkage disequilibrium estimation in rice germplasm panel was assessed. The mean SNP density was approximately one SNP at every 127 base pairs (or 8.053 SNPs/kb) across the rice genome. A total number of 2,260,030 SNPs were identified within *indica*, 1,562,078 from tropical and temperate *japonica* sub-groups and 2,933,037 from the population formed by merging *indica* and *japonica* genomic data (Supplementary note). All these quality-assured SNPs sets were used to calculate GWAS against cooked grain length, grain width, grain shape (GL_c_, GWi_c_ and GS_c_) and raw grain length, grain width, grain shape (GL, GWi and GS) within each subspecies and across the whole germplasm panel combining both subspecies. Both kinship and inferred population structure were used as covariates in a mixed linear model-based GWAS analysis. We used single-locus (SL)-GWAS approach using EMMAX for association analysis where Bonferroni corrected P-values with −log_10_P > 5 were used as a threshold criterion to fetch moderate to highly significant loci. Furthermore, we adopted the multi-locus (ML)-GWAS strategy by following three independent methods^23–25^. We detected the common highly significant hotspots validated using single and multi-locus methods on the characteristic genetic regions on chromosomes 3, 5, 7 and 11 (Table S1). Significant associations with GS_c_ were observed on chromosomes 3, 5, 7 and 10 that associated with either GL_c_ or GWi_c_ (Figs 1–3). Chromosome 3 loci (GL3.1 and GL3.1_c_, respectively) associated with GL and GL_c_ (Figs 1–3, Figs S3 and S4). While, a number of significant association signals were detected in the genomic regions on chromosome 5 (GW5) and 7 (GWi7.1/ GWi_c_7.1, GWi7.2/GWi_c_7.2) for both GWi and GWi_c_; GWi11.1 genetic region associated with only GWi. Detailed inferences on chromosome 3, 5 and 7 were presented together with indel results (see next sections).

**Figure 4:**
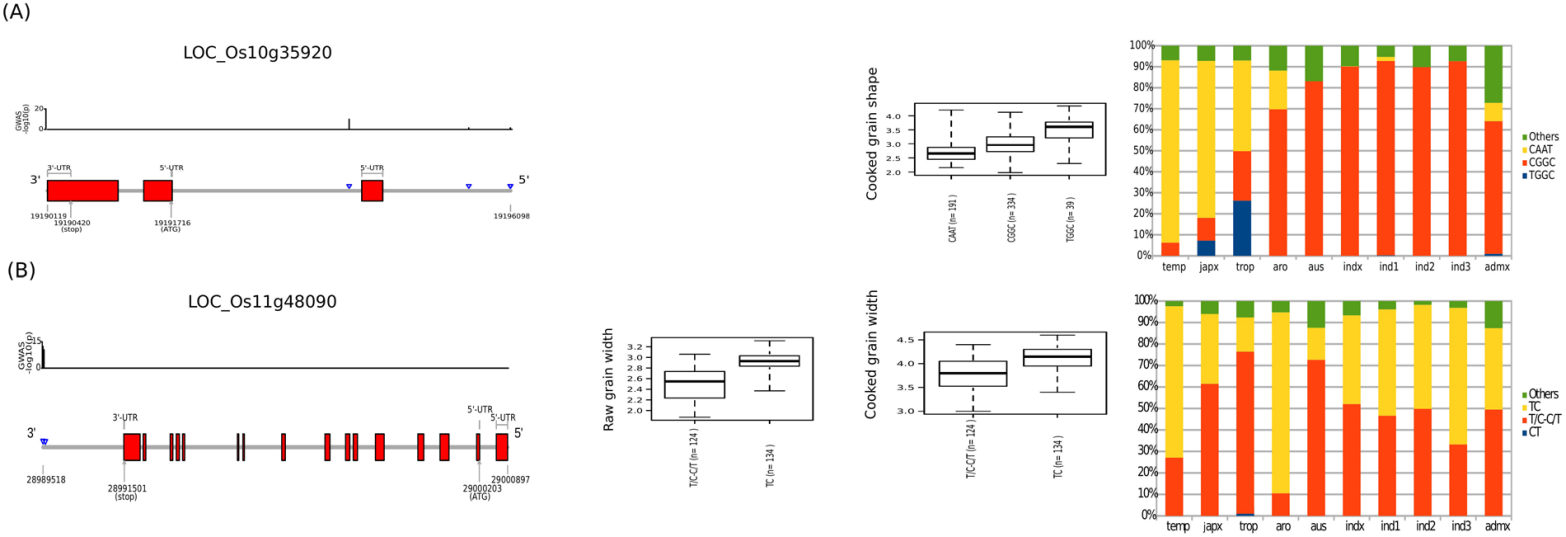
Gene structures of LOC_Os10g35920 and LOC_Os11g48090, the phenotypic variation explained by their haplotypes, and penetrance in the 3,000 rice genomes. (**A**) LOC_Os10g35920 was implicated by a single intronic SNP (GS_c_10.1) that had a significant association to cooked grain shape (GS_c_). The T allele of this SNP had the most influence in the GS_c_ variation that when combined with the G alleles of the two promoter region SNPs has the potential to result in bolder grains. Mapping haplotype TGG in the 3,000 rice genomes showed that the haplotype was present only in *japonica* accessions, particularly tropical *japonica*. (**B**) The gene structure of LOC_Os11g48090 showed the position of the significantly associated SNPs positioned at the 3′-UTR of the gene. Plotting the variation of GWi with respect to the haplotypes formed by the significant SNPs showed that the accessions that were homozygous to the T and C alleles of these SNPs showed a tendency to be bolder both in raw and cooked grains. Mapping the haplotypes in the 3,000 rice genomes showed that all haplotypes have pervasive representations in all subspecies as well as those classified as admixtures.

A GWAS peak at 28989431-29000913 bp (size = 11.5 kb) on chromosome 11 identified two novel SNPs that have highly significant associations with GWi in *japonica* but not for GWi_c_ (Fig. 1). These two SNPs, snp11_28989509 (−log_10_(*p*) = 9.3) and snp_11_28989516 (−log_10_(*p*) = 11.36), had effect of 0.94 and 1.02, respectively (Tables S1 and S2). These two SNPs were located at the 3′-UTR of LOC_Os11g48090 that was known to encode for helicase conserved C-terminal domain containing protein (Fig. 4). Two alternative haplotypes (Fig. 4B) formed by these SNPs: haplotype 1 (TC) was observed in 134 genotypes with mean GWi of ~2.9 mm, while haplotype 2 was heterozygous (T/C, C/T) and was found in 124 genotypes with mean GWi of ~2.5 mm. Mapping these two haplotypes to the 3000 Rice Genomes showed that haplotype 1 was prominent in temperate *japonica* and *aromatic* and at moderate levels in *indica*. It was under-represented in *aus* and tropical *japonica* (<18%) (Fig. 4B). Haplotype 2 appeared to have more representation in *aus*, and tropical *japonica* germplasm, moderate representation in *indica* and to a lower frequency in *aromatic* and temperate *japonica* (<30%) (Fig. 4B). Interestingly, the homozygous rare haplotype (CT) was found in extremely low percentage in tropical *japonica* (Fig. 4B).

Novel region detected by GWAS such as GS_c_10.1 from chromosome 10 associated with cooked grain shape detected only in SL-GWAS method with higher significance, where a single prominent significant SNP (C→T; effect = −0.58, −log_10_(*p*) = 9.83) was detected in the intronic region of gene E3 ubiquitin ligase (LOC_Os10g35920). Mining the potentially causal variant in 3 K RGP data suggest a limited presence of the T allele within *tropical japonica* and Japx (higher ratio of GL_c_/GWi_c_) in cooked rice grains (mean 3.48) than rest of the germplasm with C allele (mean GS 2.89) (Fig. 4A).

### Indel-based GWAS results of grain size and shape traits

A complementary GWAS analysis that used 393,429 indels highlighted associations on chromosome 3 for GL, and chromosomes 5 and 7 for GWi (Figs S5–S7). These results matched the SNP-based GWAS on their respective regions with SL (Figs 1–3) and ML approaches (Fig. S8).

**Figure 5:**
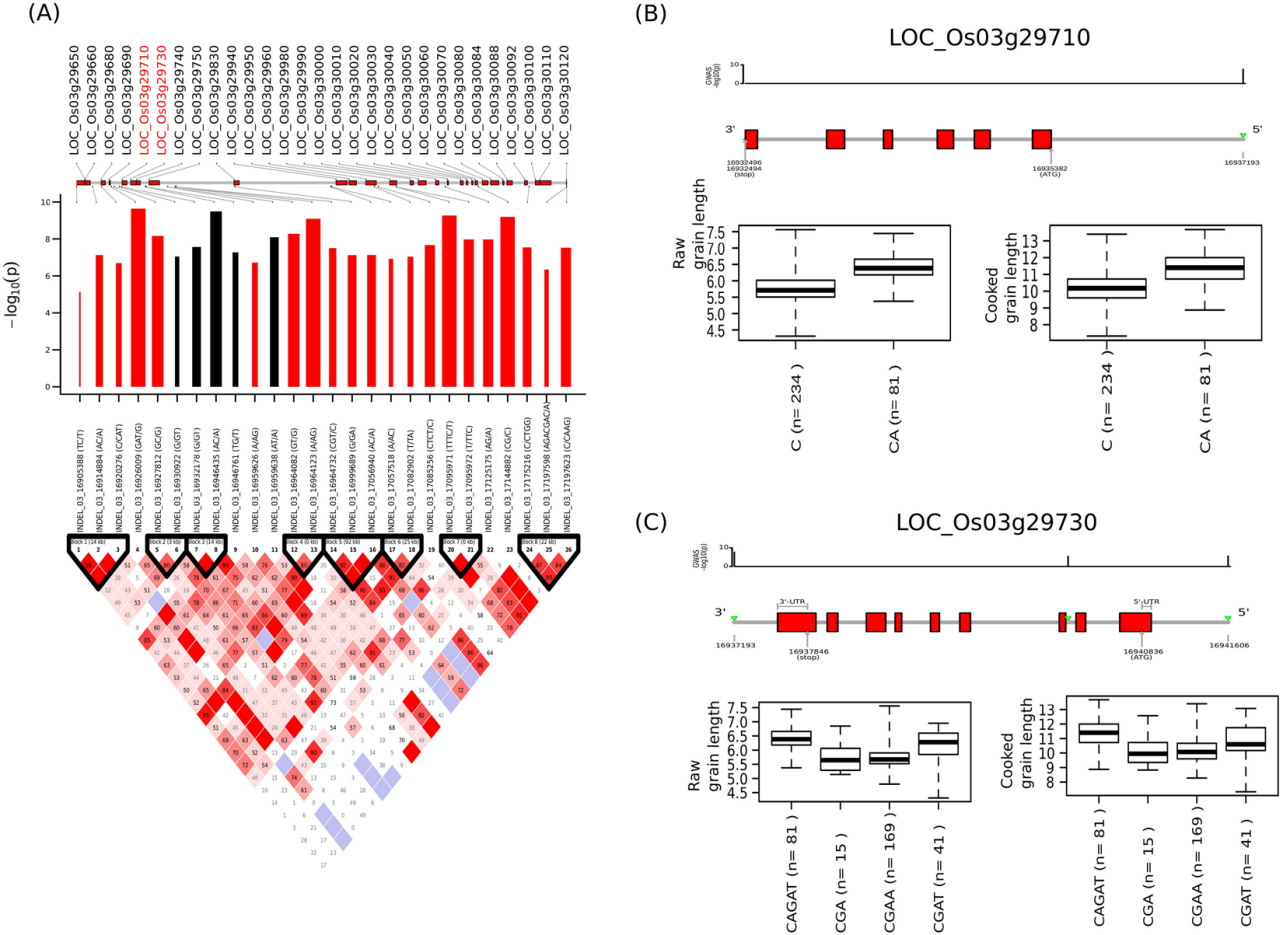
Linkage disequilibrium plot of indel association with grain length (GL) on chromosome 3 in *indica*. Linkage disequilibrium analysis using indels (**A**) revealed so much variability in this 300 kb region. Eight LD blocks were formed most not exceeding 25 kb. The bar plots show that three of the five indels with the highest likelihood of association were within the vicinity of LOC_Os03g29710 and LOC_Os03g29730. The bar plots also show that indels within the vicinity of these two genes have contrasting effects to the phenotype (black implies positive additive effect, while red is the reverse). All the others decreased grain length. The widths of the bars indicated the relative effect of the alleles. INDEL_03_16927812 and INDEL_03_16930922 were outside the genic region of LOC_Os03g27910 (**B**). Grain length distribution for both raw and cooked grains showed that CA allele of the indel associates with GL > 6.0 mm and GL_c_ > 11 mm. (**C**) Targeted association with GL and GL_c_ using indels at the control region of LOC_Os03g29730 and an intronic indel (C) showed that CAGAT was able to discriminate 81 accessions that had ~6.3 mm and ~11mm GL and GL_c_, respectively, from the rest.

The indel-based GWAS analysis revealed a 0.28 Mb region (16.90 Mb–17.20 Mb) with high linkage disequilibrium (LD) decay in chromosome 3 downstream from *GS3* that associated with GL in *indica* (Fig. 5A). Further analysis showed that haplotypes formed by indels in two genes (LOC_Os03g29710 and LOC_Os03g29730) were able to explain certain ranges in the phenotypic variation of GL (Fig. 5B,C). Interestingly, none of the significant novel allelic variants within *GS3* that explains significant associations were detected by indel-based GWAS analysis. A highly significant SNP-based GWAS association signal (−log_10_ (*p*) ≥ 17) for GL_c_ and GL were observed in a 0.42 Mb region (16.66 Mb – 17.11 Mb) on *indica* chromosome 3 (Fig. 2 and Fig. S9), mapped the topmost SNP within *GS3* gene influencing GL^9,26^. The functional SNP in *GS3* was a C→A transition (snp_03_16733441) that was previously reported^9,26^. Interestingly, this causal SNP was detected as the one most highly associating with both GL (Effect = −0.50, −log_10_ (*p*) = 26.23) and GL_c_ (Effect = −0.46, −log_10_ (*p*) = 10.41), explaining mean GL of 6.5 mm and mean GL_c_ of 11.5 mm, respectively (Table S1).

Within *indica*, a highly significant association signal (−log_10_(*p*) ≥ 18) was detected for GWi on a 0.11 Mb window (5.36 to 5.47 Mb) on chromosome 5, which was in the neighborhood region of *qSW5/GW5* (Fig. 1). This region showed up both in an indel- and SNP-based GWAS. The functional polymorphism present in *qSW5/GW5*, the region between the LOC_Os05g09510 and LOC_Os05g09520, was a 1,212 bp deletion in *japonica* reference genome (Nipponbare; MSU Release 7) that causes wide and short heavy grains^11,27^. Other major effect SNPs at the promoter region of gene LOC_Os05g09520 were also found to significantly explain variation in GWi and GWi_c_ (Fig. 1, Table S1, Fig. S10). The utility of high-resolution genotype data obtained from re-sequenced genomes showed the importance of exploring structural variations at the sub-species level to further fine-map genetic regions associated with grain quality traits as detected by SNP and indel-based GWAS.

### Haplotypes formed by novel causal variants on chromosome 7 explain variation in raw and cooked grain widths

Through SNP-based ML- and SL-GWAS analysis, prominent association signals were detected on chromosome 7 for GWi and GWi_c_ when both of the subspecies (*indica* and *japonica*) were combined. This reflected the contribution of large effect allelic combination contributed by both subspecies (Fig. 1C,F). Interestingly, less prominent association signals for GWi (−log_10_(*p*) ≥ 5) were detected on chromosome 7 within-subspecies GWAS (Fig. 1A,B,D,E). Within this broad peak on chromosome 7, two different regions [(GWi7.1/GWi_c_7.1 at intervals 22.1 Mb–22.8 Mb) and (GWi7.2/GWi_c_7.2 at interval of 23.3 Mb–25.2 Mb)], were identified to influence GWi and GWi_c_ (Fig. S11). Indel-based GWAS analysis identified 21.84 Mb–23.05 Mb that overlapped GWi7.1/GWi_c_7.1 to associate with GWi; no signal was detected at GWi7.2/GWi_c_7.2.

**Figure 6:**
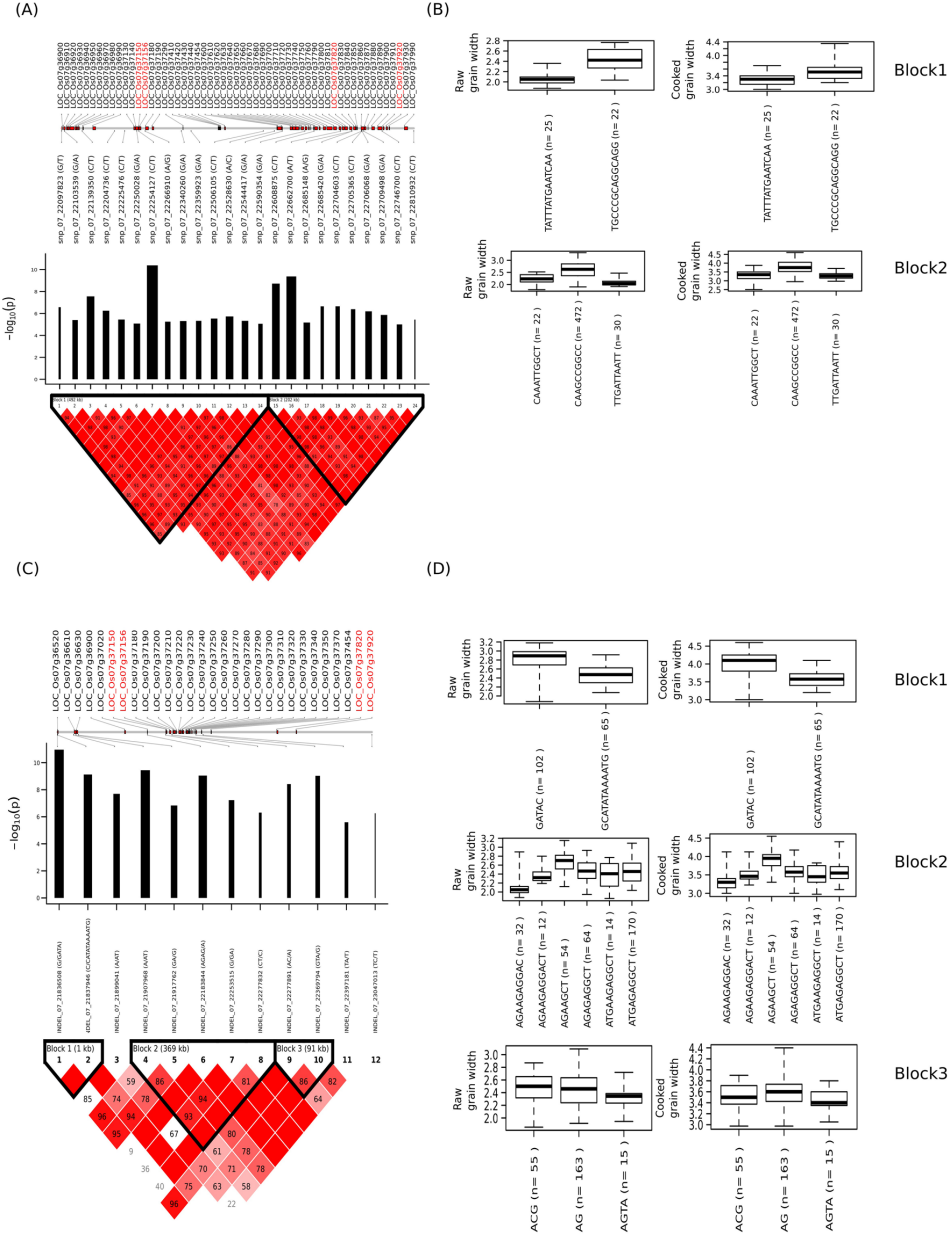
GWAS for grain width (GWi) using SNPs and indels revealed the association of a region in chromosome 7 to GWi. (**A**) The linkage disequilibrium (LD) plot of the 24 tag SNPs significantly associated with grain width. A scaled and highly dense plot of the associated genomic region on the chromosome is shown where the relevant genes are marked in red (boxes). The positions of the 24 tagged SNPs are also marked with the log_10_-scaled association P values of these 24 SNPs are shown in the bar plot where black bars reflect their relative effect sizes. The gene IDs further detected in TGAS were highlighted in red color. (**B**) Haplotypes constructed based on SNPs in LD are represented as boxplot with the phenotype values for both normal and cooked grain explained by specific haplotype. Also shown are (**C**) the linkage disequilibrium plot for indels associations for the chromosome 7 with black bar graph signifies effect size on the grain width and (**D**) Haplotype constructed with phenotype distribution within each blocks formed from the significant indels in the region represented as boxplot for both cooked and raw grain.

**Figure 7:**
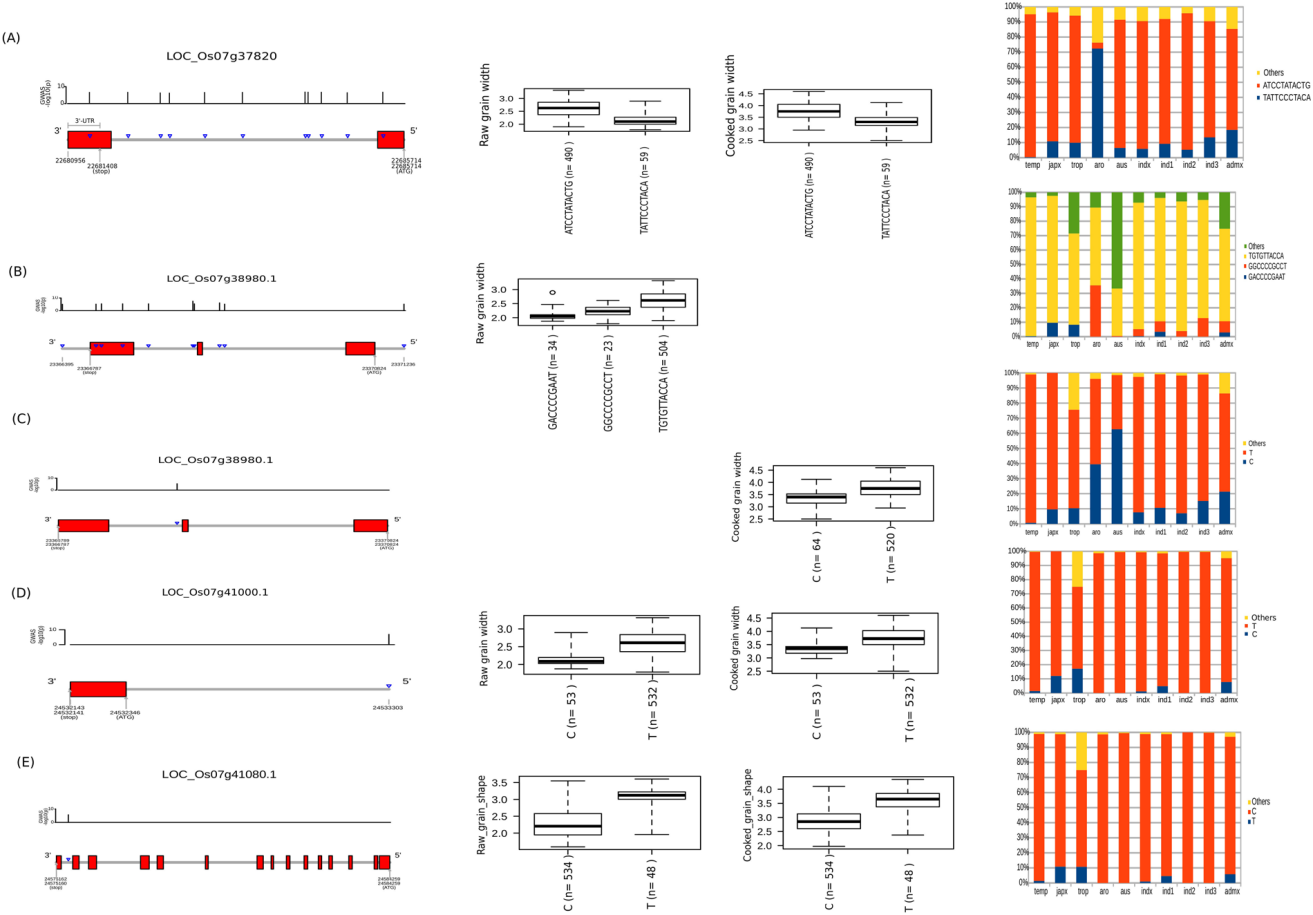
Targeted-gene association study (TGAS) in hotspot region of Chr7 (GWi7) commonly detected in single-and multi-locus GWAS identified a total of five genes that had significant association with GWi and GWi_c_. (**A**) LOC_Os07g37820 was identified as key gene in GWi7.1 region. Three additional genes (**B**) LOC_Os07g38980, (**D**) LOC_Os07g41000 and (**E**) LOC_Os07g41080 were identified through TGAS in GWi 7.2 region. The blue inverted triangles in the gene structure diagram represent the SNPs included in plotting the boxplots. Boxplots were generated to show the phenotypic distribution of haplotypes for raw and cooked GWi. Respective haplotypes mapped in 3,000 rice genome results were shown on the right panel.

A total of 24 tag SNPs at GWi7.1/GWi_c_7.1 formed two LD blocks with three unique haplotypes in each block (Fig. 6A,B, Fig. S11, Table S2). SNPs 3, 7, 15, and 16 had the strongest association likelihood and the largest allele effects among the SNPs. The indel-based GWAS results identified 12 significant structural variants with three LD blocks within GWi7.1/GWi_c_7.1 (Fig. 6C,D). The GWAS peak at this region was dissected using PLINK’s*–clump* function. Indels in block 1 and 2 showed larger allele effects compared to those indels in block 3. Indel-based targeted association of candidates within GWi7.1/GWi_c_7.1 identified 4 candidate genes (LOC_Os07g37150, LOC_Os07g37156, LOC_Os07g37820 and LOC_Os07g37920) with indels and also confirmed to possess SNP-based haplotypes that can be used to discriminate GWi. Two additional non-synonymous SNPs and an indel in 3′-UTR detected in LOC_Os07g37920 (NAC transcription factor) were found to associate with GWi (snp_07_22756160; effect = 0.22, −log_10_(*p*) = 4.15) (Fig. S13D–F). This SNP causes an amino acid change from hydrophobic glycine to polar serine. Utilizing the integrated ML- and SL-GWAS approaches, within the hotspot target region of GWi7.1, LOC_Os07g37820 was found to significantly associate with cooked and raw GWi (Fig. 7A). TGAS on LOC_Os07g37820 that included a non-synonymous SNP (snp_07_22685420; A→G) alter the protein sequence by replacing charged arginine (R) with hydrophobic glycine (G), found to associate with raw (Effect = 0.31, −log_10_(*p*) = 6.65) and cooked grain width. In addition, 10 other SNPs in untranslated and intronic regions were found in LOC_Os07g37820 (Fig. 7A, Table S1). A total of 490 accessions that had the haplotype ATCCTATACTG possessed means of 2.6 mm and 3.8 mm for GWi and GWi_c_, respectively. A total of 59 accessions that had haplotype TATTCCCTACA had means of 2.2 mm and 3.3 mm for GWi and GWi_c_, respectively (Fig. 7A).

Using the SL-GWAS approach, targeted gene association analysis in GWi7.1/GWi_c_7.1 region identified six additional loci (Fig. S13). An SNP (snp_07_22608875) lying in downstream region of the gene LOC_Os07g37710 detected to significantly affect GWi (β = 0.44, −log_10_(*p*) = 8.72) (Fig. S13A, Table S2). Low GWi haplotype TTA represented the mean GWi and GWi_c_ of 2.1 and 3.3 mm, respectively and mainly detected in tropical *japonica*, Japx and *aus* cultivars upon scanning 3000 rice germplasm. Another SNP (snp_07_22662700) present on intronic region of LOC_Os07g37790 (transcription regulator), positively associated with GWi (β = 0.51, −log_10_(*p*) = 9.37) (Fig. S13B). Similarly, low GWi haplotype T showed the mean GWi and GWi_c_ of 2.1 and 3.3 mm, respectively and detected in tropical *japonica*, Japx and *aus*. In LOC_Os07g36900 (F-box domain protein) a total of seven SNPs including three non-synonymous SNPs (snp_07_22097823, snp_07_22097824 and snp_07_22097863) were identified (Fig. S13C). These adjacent non-synonymous SNPs in exon 1 were annotated to alter the amino acid sequence by replacing charged aspartate with hydrophobic alanine. Within LOC_Os07g36900, haplotype AACATTT was found in 34 genotypes with mean GWi of 2.1 mm and GWi_c_ of 3.1 mm. Haplotype GGTGGCA was found in 508 lines with a relatively higher mean GWi and GWi_c_ of 2.6 and 3.8 mm, respectively (Fig. S13C). The identified genes associated with GWi were evaluated for their expression in low to high grain width genotypes and identified a candidate LOC_Os07g36900, differentially expressing across the lines possessing contrasting phenotypes (Fig. S15). Additionally, the moderate to high expression of gene in later growth and development stages specially in heading stage suggest their role in grain size determination (Fig. S15).

SNP based GWi7.2/GWi_c_7.2 region had three LD blocks and matched a known gene *GL7/GW7* (LOC_Os07g41200) (Fig. S12). By employing the ML- and SL-GWAS approaches, three novel candidates (LOC_Os07g38980, LOC_Os07g41000 and LOC_Os07g41080) were identified to associate with GWi and GS in target region GWi 7.2 (Fig. 7, Table S1). Within LOC_Os07g38980, GACCCCGAAT haplotype was identified for fixing and retaining the lower GWi in tropical *japonica*/japx and aromatic lines (Fig. 7B,C). Upon TGAS, intronic-SNP (snp_07_23368244; C→T), detected in novel gene LOC_Os07g38980 encoding unclassified protein significantly associated with raw and cooked GWi (Fig. 7B,C). Throughout the genome ML-GWAS independently yielded a number of candidates associated with cooked and raw grain dimension traits (Table S3). In addition, ML-GWAS also identified and confirmed the loci detected with less significant critical value using SL-GWAS, owing to stringent correction criterion (Table S3).

### Mapping grain size and shape genes to syntenic map created between *japonica* and *indica* reference genomes

**Figure 8:**
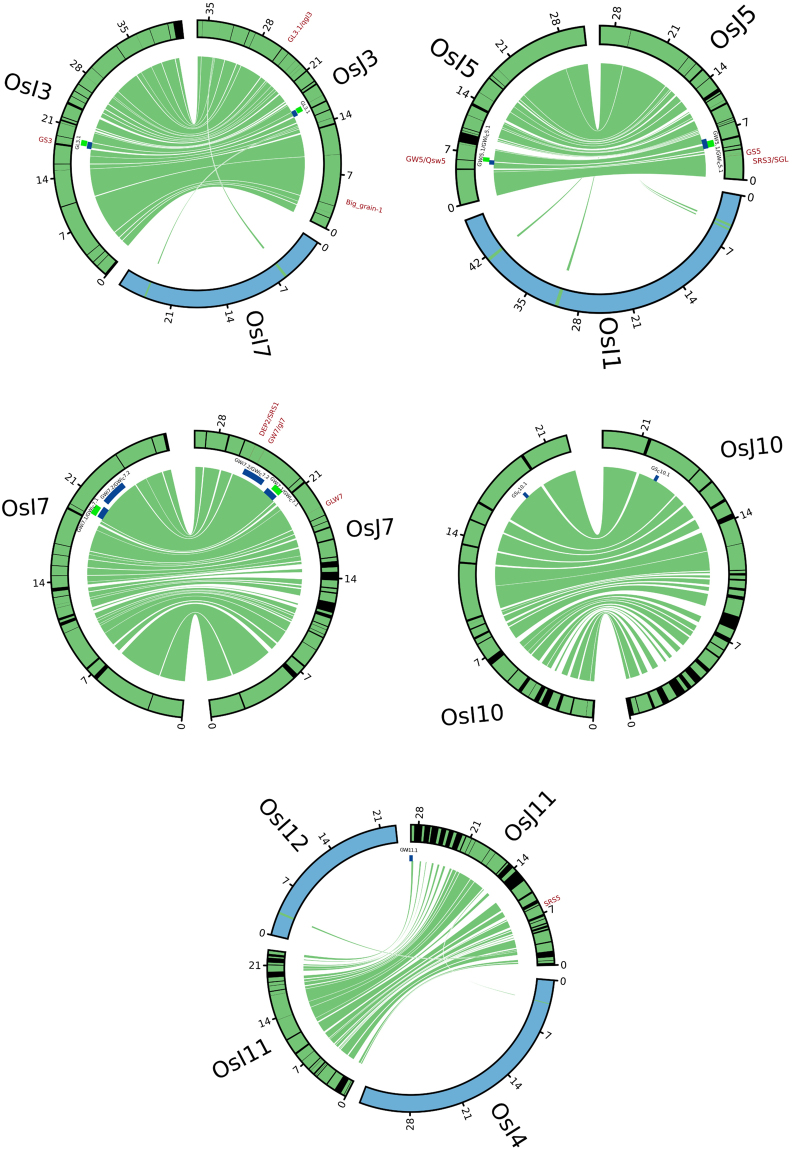
Detailed sequence collinearity in chromosomes 3, 5, 7, 10 and 11 between *indica* and *japonica* reference genomes. Circos plots showed syntenic relationship of five chromosomes (physical size shown in Mb) within *indica* and *japonica* subspecies based on protein sequence alignment. Conserved regions were shown in green while black lines represented the break points. The positions of genes that have been cloned for grain width, length and shape were also labeled in the synteny map. Blue and green boxes represented regions in the respective chromosome where GWAS peaks were detected; blue were for SNP-based GWAS, while green were for those done using indels. It was evident that most of the cloned/characterized genes for grain size and shape were in collinearity between *indica* and *japonica*.

In order to dissect the degree of structural variation, the syntenic relationship at subspecies level was evaluated by doing comparative genomics between Nipponbare (MSU Release 7) and 93-11, reference genomes of *japonica* and *indica*, respectively. We found that synteny was high for chromosomes 3, 5 and 7 with apparently several random break points occurring throughout these chromosomes (Fig. 8). Chromosome 11 depicted severe erosion in collinearity. We have noted significant SNP- and indel-based GWAS association signals on chromosomes 3, 5 and 11 that occurred at synteny break points (Fig. 8). GL3.1, a region that has an uncharacterized gene at a synteny break point in chromosome 3, was detected by indel-based GWAS to be significantly associated with GL. For this trait, the most significantly associated SNP detected by a SNP-based GWAS was from the cloned GS3 gene found at the conserved region of chromosome 3 of *indica* and *japonica*. For GWi and GWi_c_, we found an association signal at GWi5.1 on chromosome 5 that confirmed the effect of the deletion upstream of *GW5* in *japonica* that is absent in *indica*. *GW5* was implicated by several significantly associated SNPs at its promoter region. It is interesting to find neighborhood of major GW regulating gene *GW5* ^27^ at the collinearity break between sections of chromosomes 5 in both *indica* and *japonica*. We also similarly fine-mapped GWi11.1 that matched to a collinearity break point in chromosome 11 significantly associated with grain size and shape (Fig. 8). Significant association signals were exclusively detected on chromosome 11 in *japonica*. The break points in chromosome 3, 5 and 11 of *japonica* were due to structural variations such as insertion or deletion (Fig. 8). These structural variations at the subspecies level led to the loss of collinear regions within *indica* and *japonica* subspecies.

It is interesting to note that many of the cloned genes for grain size in rice were found to match the collinear regions in the syntenic map (Fig. 8). TGAS for known genes which were previously cloned for grain dimensions and shape enabled the detection of additional alleles and confirmed previously reported SNP variants, though the respective association likelihoods were weak to moderate (Table S4, Fig. S14). The allelic variations at the subspecies level that lie within *Gif*1, *GL3*.*1*, *big grain2*, and *short grain1*, genes that were known to regulate grain size and shape in both *indica* and *japonica* ^6,28^, could likely be attributed to preferential selection of alleles in both subspecies during domestication. The allelic variations in *An-1*, *GS5*, *small grain11* were preferentially enriched in *japonica*, while *GL7/GW7*, *small grain1*, *srs5* allelic variation were enriched in *indica* subspecies (Table S4, Fig. S14).

## Discussion

Grain dimensions are major determinants of grain weight and therefore are important component traits affecting crop yield. These traits also influence varietal acceptability to consumers, and therefore rice grain size/shape is a major preferential target trait in breeding^5,29^. Short and bold type cultivars are highly preferred by many consumers in Japan, South Korea and northern China, whereas consumers in India, the USA, and other South and Southeast Asian countries favor long slender and medium slender grains^30^. Many of the genes/QTLs that regulate grain length, width and thickness function in selective proteolysis, as well as those that promote cell proliferation and expansion have been cloned, characterized and further validated in populations developed either from crossing within or across the subspecies (Table S4). These traits have been shown to be controlled by both major and minor genes or QTLs that often exhibit additive effects, dominant effects or both^31^.

Previously, moderate to high coverage SNP genotype data generated through genotyping by sequencing and Affymetrix array-based genotyping platforms were used to perform GWAS analysis and to decipher the genetics of rice grain length^7^. In the present study, high-density genotype data derived from re-sequenced genomes of 591 diverse landraces were used to identify the novel allelic variants and the haplotypes that underlie grain length, width and shape in raw and cooked rice grain. A total of ~2.9 million high-quality SNPs and 393,429 indels were used as the genotype resources in complementary SNP- and indel-based GWAS analyses. These genotype data densities and the substantial level of population divergence and within-population genetic variation evident in *indica* and *japonica* make it highly likely for GWAS to catch trait-associated alleles. The resolution provided by the dense SNP markers was at least three-folds higher than the recently published reports on GWAS in cultivated rice^7,8,18^. This clearly reflects the advantage of high-coverage re-sequencing based SNP genotyping resources in *indica* population where LD decays faster (Supplementary Note) compared to *tropical* and *temperate japonica* ^5,32^. This study enabled the discovery of large numbers of SNPs and indels that differentiate the important traits especially cooked rice grain dimensions and shape that are being selected during domestication. For cooked grain, a distinct allelic variant was revealed in the intron region of gene encoding E3 ubiquitin ligase on chromosome 10, exerting substantial effect on cooked grain shape. Other E3-ubiquitin ligases were earlier revealed to regulate the grain width in rice^33^ and grain size in other crops^34^.

GWAS analysis performed on a genome-wide SNP matrix that was designed to cover coding as well as the regulatory regions such as promoters, alternative spliced junctions, 5′ and 3′ untranslated region helped in the discovery of novel regulatory alleles and their haplotypes. Genetic dissection of the genomic region detected on chromosome 11 in *japonica* revealed the presence of two adjacent SNPs (7-bp apart) in the 3′-UTR region of LOC_Os11g48090, a gene that encodes helicase conserved C-terminal domain containing protein in rice (Fig. 4C). These SNPs showed strong associations with grain width. Interestingly, none of the other SNPs in this region were implicated by LD, to influence grain width. In addition, a short region containing causal SNPs present within 3′-UTR showed ~90% homology with the binding site of orthologous members of High Mobility Group (HMG) Box DNA-binding proteins (namely AHL12 and AHL25), previously known to have a role in Arabidopsis growth and development^35–38^. Transcriptional repressors target the 3′-UTRs and the region near the stop codon^39^. Therefore, nucleotide substitutions noted in the 3′-UTR region of gene LOC_Os11g48090 potentially lead to the disruption of the binding site of a repressor. This requires further functional validation. The haplotypes comprised of these causal SNPs that are responsible for conferring wider grain width (mean = 2.9 mm), were detected in *temperate japonica*, and heterotic alleles T/C-C/T explaining lesser grain width (mean = 2.5 mm) abundantly represented in *tropical japonica* and *aus* (Fig. 4). The haplotype T/C or C/T representing heterozygous region for this allele in inbred background lines, which explains lower grain width represent the contribution of heterogeneous inbred families. This supports the general phenotypic observation that *temperate japonica* accessions possess wider grain width compared to *tropical japonica* ^6,13,14^.

Genomic analysis on global MAGIC populations, developed specifically to explore effects of allelic recombination between *japonica* and *indica* subspecies, was able to identify a region located at position 22.7 Mb – 26 Mb on chromosome 7 that significantly associates with grain width^40^. GWAS in this study performed in a combined *indica* and *japonica* populations was able to identify GWAS peaks as GWi7.1 and GWi7.2 on chromosome 7 which have significant distance to affect independently the grain width (Fig. 1, Fig. S11). This region overlaps with what was previously reported for raw grain using the MAGIC population^40^ and bi-parental population^21^. This also suggests that functional allelic variations present in the two major subspecies were highlighted effectively when combined. Our study further narrowed down this major effect region into two sub-regions with GWi7.1 mapped at the interval of 22.1–22.8 Mb and GWi7.2 narrowed down to a region of 23.3–25.2 Mb. Employing integrated multi-locus and single-locus GWAS approach (EMMAX) led to verify the significance of underlying target regions, GWi7.1 and GWi7.2 and simultaneously identify novel candidate genes. Furthermore, TGAS of the GWi7.1 region identified four putative candidate genes that are significantly associated with grain width. Among these, one encodes for NAC-transcription factor and three are unknown protein encoding genes that possess indel haplotypes and non-synonymous SNPs differentiating cooked and raw grain width phenotypes (Fig. 7, Fig. S13). Identification of NAC transcription factors as candidate genes is coherent with the reported role of another orthologous NAC transcription factors for rice grain size^41^. For the GWi7.2 region, major effect loci representing novel variations were spotted along with previously characterized loci within *GL7/GW7* that were known to regulate grain length/width^42^ and identified 3 additional genes (LOC_Os07g38980, LOC_Os07g41000 and LOC_Os07g41080) influencing grain width. Employed multi-locus GWAS^23–25^ was helpful to validate novel loci identified from the genomic regions using single-locus GWAS method with a less stringent significance criterion. The present study demonstrated the power and resolution of whole genome re-sequencing to identify novel genomic variants and their haplotypes that contribute significantly in providing and fixing narrower grain width for both raw and cooked rice grains (~2.0 mm/~3.0 mm respectively).

Many of the cloned size and shape related genes comprising *GW8/OsSPL16*, SRS5 and TGW6 were mapped in conserved regions^7,43–45^. From high-resolution SNP- and indel-based GWAS, several significant associations that mapped to synteny breaks were revealed in this study. These findings show how structural variation influences grain size traits. In the *indica* population, the GL3.1 association signals for grain length on chromosome 3 (16.12–16.40 Mb) and GW5.1 detected on chromosome 5 (5.36–5.47 Mb) grain width were mapped to the break points. Comparative genomics to study synteny in this region unveiled extensive sequence collinearity with intermittent gaps within gene contents, intergenic regions and gene orders across *indica* and *japonica* reference genomes. This was consistent with the previous reports on rice genome diversity^46–49^. The breakage in synteny was confirmed due to several significant SNP variation and deletion detected upstream of cloned *GW5* region influencing grain shape/size. The rearrangements leading to insertions and deletions caused larger synteny breakage relative to others, for instance, in case of upstream of *GW5*, a 1212 bp region was deleted in Nipponbare genome (*japonica*) corresponding to ~2300 bp fragment of Kasalath genome (*indica*)^7,27,50^. Although several important alleles explaining major QTLs for grain dimensions and shape in raw rice grains were confirmed in *GS3* and *GW5* genes^26,27,50,51^, this study has identified additional causal indels particularly in GW3.1. Additionally, the GWAS peak identified for GWi11.1 found only in *japonica* overlapped a break region.

Using structural variants as genotype data in GWAS offered new insights to map novel alleles located in the break regions that influence grain size and shape. The use of *japonica* reference genome in this study to account for indel variation across *japonica* and *indica* sub-species was informative and effective in identifying novel genes located at regions where the synteny between these two subspecies breaks. For future studies, taking the subspecies level structural variations into account by mapping the reference genomes and calling SNPs using a much improved *indica* reference genome^52,53^ could further enhance the precision and resolution of the genome-wide mapping studies at the subspecies level. In this regard, utilization of the recently released map-based high-quality genome sequence and annotations of major varieties (Zhenshan 97 and Minghui 63) of *indica* as a reference^54^ could potentially lead to even more profound understanding of the genetics of certain traits.

A novel approach implemented in the present study, where the result of comparative genomics between reference genomes of two major *Oryza sativa* L. sub-species used in conjunction with GWAS using whole genome re-sequencing resources to identify structural variation in genes influencing cooked and raw grain size and shape in rice. Through this pipeline: (1) Significant association signals that implicated novel genes associated with cooked and raw grain width, length and shape for each subspecies were identified through SNP- and indel-based GWAS analysis; (2) Diagnostic haplotypes were defined through LD analysis and tag SNP approaches; (3) The syntenic maps were derived between the subspecies and mapped the GWAS peaks to identify genetic regions which falls in collinear break points, and conducted targeted associations to reveal novel gene based haplotypes; (4) Mining the 3000 Rice Genomes for the rare haplotypes found in this study revealed rare germplasm that fall into specific grain width and length ranges in each *Oryza sativa* L. sub-species. This study has built a series of genomic pipelines that expedited the identification of novel grain dimensions and shape genes and also provided a comprehensive understanding of the distribution of alleles and haplotypes that are deemed preferentially selected for grain dimensions and shape for both raw and cooked grains though the process of domestication.

## Methods

### Plant materials

A total of 591 diverse accessions whose days to maturity do not exceed 140 were selected from the 3000 resequenced rice genomes^22^ (Table S5). This panel was comprised of 324 *indica* and 267 *japonica*. They were grown in a one and a half hectare contiguous experimental area at the International Rice Research Institute (IRRI), Laguna, Philippines (14°N, 121°E) during the 2015 dry season. Complete block design was used to group lines whose days to maturity were as close as possible to prevent confounding effects. Blocks were designed to accommodate 80 accessions. These accessions were randomly positioned within their respective blocks, and the blocks randomly positioned in each of the four replicates in the case of *indica*, and two replicates in the case of *japonica*. The dimension of each rectangular block was 12.5 m × 50 m. Uniform field and crop management procedures based on IRRI standard practices were followed across all replicates. Manual harvesting was done when seeds reached the optimum moisture content (MC) (22–24%), and subsequently hand-threshed to avoid physical trauma to the seeds. Standard IRRI drying method was followed until seeds attained 12–14% MC. Seeds were then stored in brown double-layer seed paper bags at seed storage room where temperature was maintained at 18 °C.

### Phenotyping for measurement of grain dimensions

Quantities of 50 g seed material from every line with independent replicates were obtained and subsequently equilibrated at room temperature before any physical analysis was done. For cooking, rice grains were cooked at 100 °C using the standard operating procedure of IRRI Grain Quality and Nutrition Service Laboratory (GQNSL). Grain size and shape were measured using the ISO 17025 certified protocols of the IRRI GQNSL that used a SeedCount SC5000 Image Analyser (http://www.knowledgebank.irri.org/ricebreedingcourse/bodydefault.htm#Grain_quality.htm).

### Genome-wide SNP identification

A total of 591 diverse landrace accessions composed of 324 *indica* and 267 *japonica* varieties were used for the genome-wide SNP identification. Publicly available variant call format (VCF) files from the published 3,000 rice genomes^22^ were used to compile the SNPs. These VCF files contained the complete base calls across the entire genome from which SNPs and INDELS were identified. Each VCF file was filtered using VCFtools^55^ to keep only those base calls with a Phred score of 30 or better. After filtering, the quality assured VCF files were then merged, all SNP and INDELs were then extracted separately, saved into two different VCF files for SNPs and INDELS, and then subsequently converted into PLINK^56^ format (BIM, BED, FAM).

### Population structure and calculation of linkage disequilibrium decay

Principal components analysis (PCA) was performed in diversity lines using the SNPRelate package in R to detect the population structure^57^. An LD cut-off of 0.99 was used that resulted in the selection of 673,846 SNPs in PCA calculation. The first two principal components accounted for 42.57% of the total genetic variation. For linkage disequilibrium (LD) decay prediction, we calculated the pairwise LD of all SNPs present in the three different populations using PLINK v1.90 beta (PLINK2). Then, bins representing multiples of 50-kb distances between SNPs were formed where the mean r^2^ for each bin calculated using a custom PERL script and plotted. This was done for each sub-population.

### GWAS analysis

The genotype file was filtered prior to running GWAS analysis. Using plink2, we retained individuals and SNPs that had a missing rate of not more than 5% and then filtered for a minor allele frequency of at least 5%. This filtering step resulted to a final set of 2,933,037 SNPs and 585 distinct varieties from both *indica* and *japonica* subspecies. *Japonica* accessions accounted for 1,562,079 total numbers of SNPs from 267 individuals, while 324 indica lines accounted for 2,260,030 SNPs. A total of 393,429 indels were considered after filtering with missing rate of not more than 5% and then filtered for a minor allele frequency of at least 5%. Phenotype data was then transformed using WarpedLMM^58^ to satisfy the data distribution requirement of mixed linear model separately for SNPs and INDELs. WarpedLMM uses a monotonic warping function to transform the phenotype data where instead of using a static function it searches for a most suitable transformation function for the given phenotype data. EMMAX^59^ was used for computing the single-locus association statistics where the kinship and population structure were added as covariates into the mixed linear model for both SNPs and INDELs. The statistical model underneath the EMMAX uses variance component model as described in the Kang *et al*.^59^ (See Online Methods of the paper for the complete details) that belongs to the family of mixed linear models. This approach to GWAS effectively corrects the confounding factors such as relatedness between samples and population structure that if uncorrected would lead to spurious associations. In this model, the predictors were composed of 1.5, 2.2 and 2.9 million SNPs for *japonica*, *indica* and combined population, respectively along with the identity-by-state kinship matrix and the population structure computed using principal components analysis. The markers were considered as fixed effects, while the kinship matrix and principal components were treated as random effects. We assumed that each term is normally distributed, although EMMAX did not explicitly mention the requirement. Beta-coefficient, indicating the effect size of the marker on respective phenotype, was mentioned as ‘effect’ in the outputs of GWAS.

Kinship was calculated using emmax-kin while the population structure was represented using the first two principal components. IBS matrix was used as kinship matrix. Two principal components were sufficient covariates for the combined *indica* and *japonica* set, as well as the *indica*-only set, while three principal components were required for the *japonica* set. These decisions were based on the scree-plot derived from the PCA results. The statistical model used for GWAS was a variance component model reported in the Kang *et al*.^59^. The threshold value was set at 1.70e-08 using Bonferroni correction (or −log_10_(0.05/2933037) = 7.77 as shown in the Manhattan plot) for identifying the peak association signals, however, a LD-based tagged SNP criteria was followed on SNPs with −log_10_P > 5 for detailed analysis on raw grain phenotype. The complete parameters when clumping for SNPs and INDELs (in plink2) were –clump-p1 1e-7, –clump-p2 1e-5, –clump-kb 200, and –clump-r2 0.5. These parameters ensures that the “index” SNPs around which the “clumps” were formed must have a p-value of at most 1e-7, while those SNPs forming the clump around the index SNP must have a p-value of at most 1e-5. Due to less significant SNPs–clump-p2 parameter was considered as 0.01 (p-value) in case of cooked grain width by following the clumping method outlined in our previous study^60^. The farthest SNP that may be clumped to the index SNP was 200 kb with an LD r^2^ = 0.5. All genomic positions and gene annotations were based on the Nipponbare reference genome (MSUv7). Tag SNPs were identified using Haploview for groups having an LD coefficient D’ ≥ 0.8, while haplotype blocks were also formed from the same result^61^. In-house R-scripts were used for creating −log_10_ (*p*) value plots and box plots for depicting phenotype distribution within the designated haplotypes. Targeted-association study was performed where only genic SNPs and those falling within 2 kb upstream and 1 kb downstream of the genic region. After TGAS we plotted the significant SNPs showing causal association with phenotype (log_10_(*p*) value). Non-synonymous SNPs were determined upon completing SNP annotation using annovar.

### Multi-locus GWAS analysis

We reanalyzed the GWAS on a same set of population earlier conducted through single-locus (SL)-GWAS, using three different multi-locus (ML) GWAS tests–FASTmrEMMA^25^, mrMLM^24^ and ISIS EM-BLASSO^23^. SNP pruning was performed on the entire SNP set (mentioned above) since memory constraint was observed with entire SNP set to run MLA tools. Therefore, a window size of 5 Kb (to include more SNPs in the analysis), a step size of 5 SNPs in each step and r^2^ = 0.5 were used as the pruning parameters in PLINK. Finally, with the 1393842 SNPs (combined *indica* and *japonica*), 1079207 unique SNPs for *indica* and 586697 unique SNPs for *japonica* were extracted and were directly used for conducting ML-GWAS using all of the three aforementioned methods. Default critical *p-*value criterion was adopted as per the details mentioned in respective method. Two principal components were used for both *indica* specific GWAS and for the combined set of *indica* and *japonica*, whereas three principal components were used for *japonica* population. Manhattan plots were created using the first step result of the multi-locus association. For tabulation of the SNP loci from all of the three methods, LOD ≥ 3 were considered as threshold parameter. The genomic regions surpassing the threshold significance criteria of LOD ≥ 3 (in case of ML-GWAS) and −log_10_P ≥ 5 were considered as common regions between ML-and SL-GWAS methods. The genetic regions simultaneously and individually identified in ML-and SL-GWAS were further categorized in respective Tables S1–S3.

### Transcriptome/expressional analysis

The expression profiling across different stages of plant growth and development was determined by using Affymetrix rice genome array at public database Genevestigator^62^. The log2 transform values were utilized to construct a plot. For differential expression profiling, total RNA from selected lines was extracted from developing (16 d post antithesis, dpa) grains for transcriptome analyses utilizing a genome-wide microarray platform (Agilent Technologies) (methods adapted from^60^). The gene expression profiling was conducted by hybridizing onto a genome wide microarray slide for rice based on the manufacturer’s protocols (Agilent Single Color; Agilent Technologies).The data was normalized using GeneSpring GX (Agilent, Santa Clara, CA) following quantile normalization algorithm. Lines with contrasting phenotypes were selected for expression analysis and the log2 transform value has been shown as heat map using Genesis tool^63^ with variance of ±3.

### Mapping out the haplotype blocks to the rest of the 3,000 Rice Genomes

SNP-seek database^64^ was used to determine the enrichment of the phenotype discriminating haplotype blocks in each of the different subspecies represented in the complete 3,000 rice genomes panel. The distribution representation of the haplotype blocks in each subspecies was calculated and visualized as percentages.

### Synteny overlays with grain size and shape genetic regions

We used reference protein sequences of *japonica* and *indica*, adapted from reference genomes of MSU version 7 and gramene database, respectively. We followed the all-to-all blastP of respective protein sequence using NCBI-BLAST-2.2.28+ tool^65^. A stringent criterion of e-value of e-30 was used in blast alignment of protein sequence within *japonica* and *indica* considering their genetic similarity at sub-species level. Subsequently, collinearity was established using MCScanX^66^ with the threshold of 10 genes constructing each collinear block. On the basis of these outcomes, colinearity was identified and represented in the form of circos^67^ that further overlaid with genomic region corresponding to cloned and characterized genes regulating grain size and shape and significant genomic region regulating grain width detected as an outcome of GWAS.

## Electronic supplementary material

Supplementary note, Supplementary Figures S1-S15, Supplementary Table S4

Supplementary Table S1, Supplementary Table S2, Supplementary Table S3 and Supplementary Table S5
