# Supplementary material for: Whole genome sequencing-based association study to unravel genetic architecture of cooked grain width and length traits in rice
Source: Sci Rep. 2017 Sep 29;7:12478. doi: 10.1038/s41598-017-12778-6 (PMC5622062; doi:10.1038/s41598-017-12778-6)
Supplement: Supplementary file 1 — Supplementary note, Supplementary Figures S1-S15, Supplementary Table S4 [file 41598_2017_12778_MOESM1_ESM.pdf]

Whole genome sequencing based association study to unravel genetic architecture of cooked grain width and length traits in rice

Gopal Misra[1], Saurabh Badoni[1], Roslen Anacleto[1], Andreas Graner[3], Nickolai Alexandrov[2] and Nese Sreenivasulu[1]*

[1]Grain Quality and Nutrition Center, Plant Breeding Division, International Rice Research Institute, DAPO Box 7777, Metro Manila 1301, Philippines
[2]Genetics and Biotechnology Division, International Rice Research Institute, DAPO Box 7777, Metro Manila 1301, Philippines
[3]Leibniz institute of Plant Genetics and Crop Plant Research (IPK), Corrensstrasse 03, 06466 Gatersleben, Germany

Supplementary Note

High-throughput genotyping data derived based on whole genome sequencing information

We harnessed the single nucleotide polymorphism (SNP) variation by aligning the genotypic data of the *japonica* Nipponbare reference genome with the high-quality re-sequencing data generated from 591 accessions enriched with *indica, temperate* and *tropical japonica* subspecies that represents the global genetic diversity originating from 72 countries[1]. After SNP calling, we have identified a total of 2,933,037 high-quality SNPs with a total genotyping rate of 98.89%. A total number of 2,260,030 and 1,562,078 SNPs segregated within *indica* and *japonica* (tropical and temperate *japonica*) sub-group, respectively, while 889,071 were common SNPs. The mean SNP density was approximately one SNP at every 127 base pairs (or 8.053 SNPs/kb) across the rice genome. This value is comparable to reports from other previously published studies that worked on re-sequencing data [2-4] and higher than array-based genotyping methods [5-7].

Genetic structure and linkage disequilibrium estimation in rice germplasm panel

Principal component analysis (PCA)[8] was carried out to summarize the global genetic variation present in selected panel of 591 accessions based on 2.9 million high quality SNP data matrix (Fig. A). The first two principal components (PC) explained 42.57% of total genetic variation, 38.24% was from PC 1 and the remaining 4.33% was from PC 2. These two principal components were able to show the genetic differentiation of subspecies present in the germplasm. Based on an unsupervised clustering of germplasm that arose from dimension reduction it was observed that *indica* and *japonica* were majorly represented. The *japonica* subspecies subdivided further into *temperate japonica* and *tropical japonica*. Few represented lines from *aus* subpopulation were interspersed between *indica* and *japonica* subgroups. The calculated mean pairwise population differentiation (*Fst*) value between both subspecies was 0.46, which confirmed the distinct diversity present at the subspecies level among these diverse germplasm. These calculations have shown that we have captured a representative genetic diversity from the original set of 3,000 rice genomes setting the stage right for GWAS analysis. Linkage disequilibrium (LD) decayed more rapidly in *indica* than in *japonica* confirming an expected wider genetic diversity in *indica* compared to that in *japonica* (Fig. B) as is currently known. This was similarly shown by sequence diversity coefficients (π) of 0.0025 and 0.0022 in *indica* and *japonica*, respectively, which were higher than the previously published values of 0.0016 and 0.0006, respectively[2].

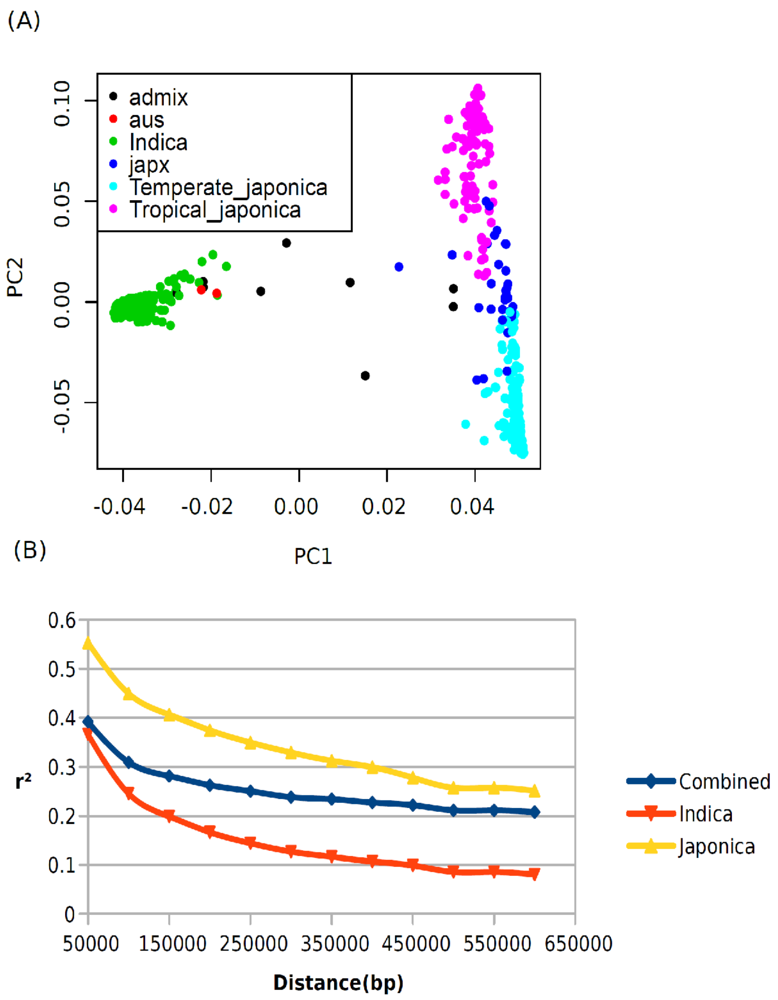

Figure: Genetic structure and linkage disequilibrium (LD) estimation in 591 germplasm (A) Principal component analysis (PCA)-plot of the first two components of 591 rice accessions; PC1 and PC2 represented the genetic variation of 38.24% and 4.33% with a total of 42.57%, present in germplasm set. (B) Genome-wide LD decay estimated from *indica*, *japonica* and combined population.

Supplementary References

1 The 3000 Rice Genomes Project. The 3,000 rice genomes project. *Gigascience* **3**, 7, doi:10.1186/2047-217X-3-7 (2014).

2 Huang, X. *et al.* Genome-wide association studies of 14 agronomic traits in rice landraces. *Nature Genet.* **42**, 961–967, doi:10.1038/ng.695 (2010).

3 Huang, X. *et al.* Genome-wide association study of flowering time and grain yield traits in a worldwide collection of rice germplasm. *Nature Genet.* **44**, 32-39, doi:10.1038/ng.1018 (2012a).

4 Huang, X. *et al.* Genomic analysis of hybrid rice varieties reveals numerous superior alleles that contribute to heterosis. *Nature Communications* **6**, 6258, doi:10.1038/ncomms7258 (2015).

5 Zhao, K. *et al.* Genome-wide association mapping reveals a rich genetic architecture of complex traits in Oryza sativa. *Nature Communications* **13**, 467, doi:10.1038/ncomms1467 (2011).

6 Crowell, S. *et al.* Genome-wide association and high-resolution phenotyping link Oryza sativa panicle traits to numerous trait-specific QTL clusters. *Nature Communications* **7**, 10527, doi:10.1038/ncomms10527 (2016).

7 McCouch, S. R. *et al.* Open access resources for genome-wide association mapping in rice. *Nature Communications* **7**, 10532, doi:10.1038/ncomms10532 (2016).

8 Price, A. L. *et al.* Principal components analysis corrects for stratification in genome-wide association studies. *Nature Genet.* **38**, 904-909 (2006).

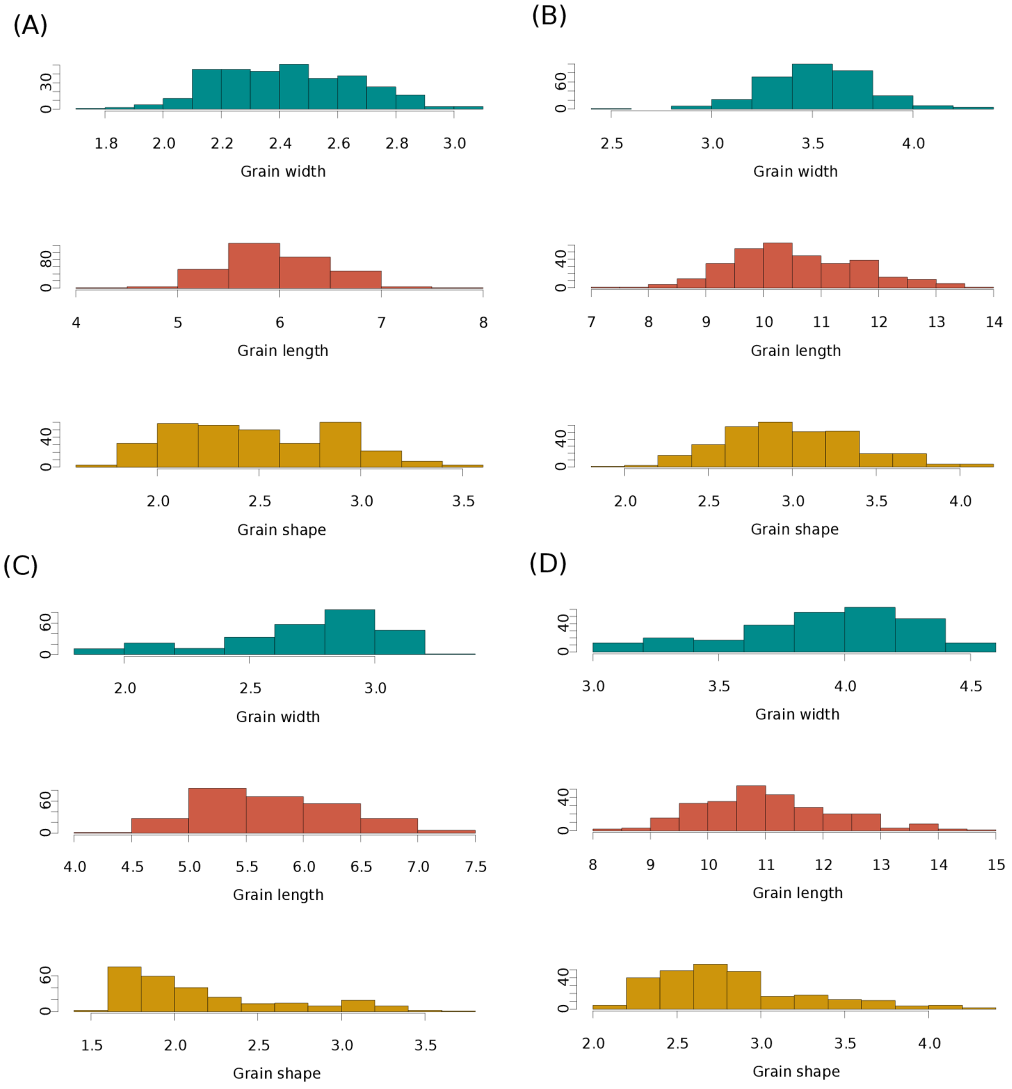

Figure S1: Phenotypic variation for -raw and -cooked grain width, grain length and grain shape in *indica* and *japonica*. Grain dimensions in *indica* in -raw(A) and -cooked grain(B); grain dimensions in *japonica* in –raw (C) and -cooked grain (D). X-axis represents respective trait value (in millimeter) and y-axis represents number of samples. All traits showed the normal distribution pattern except in case of grain width and length of *japonica*, where skewed distribution was observed.

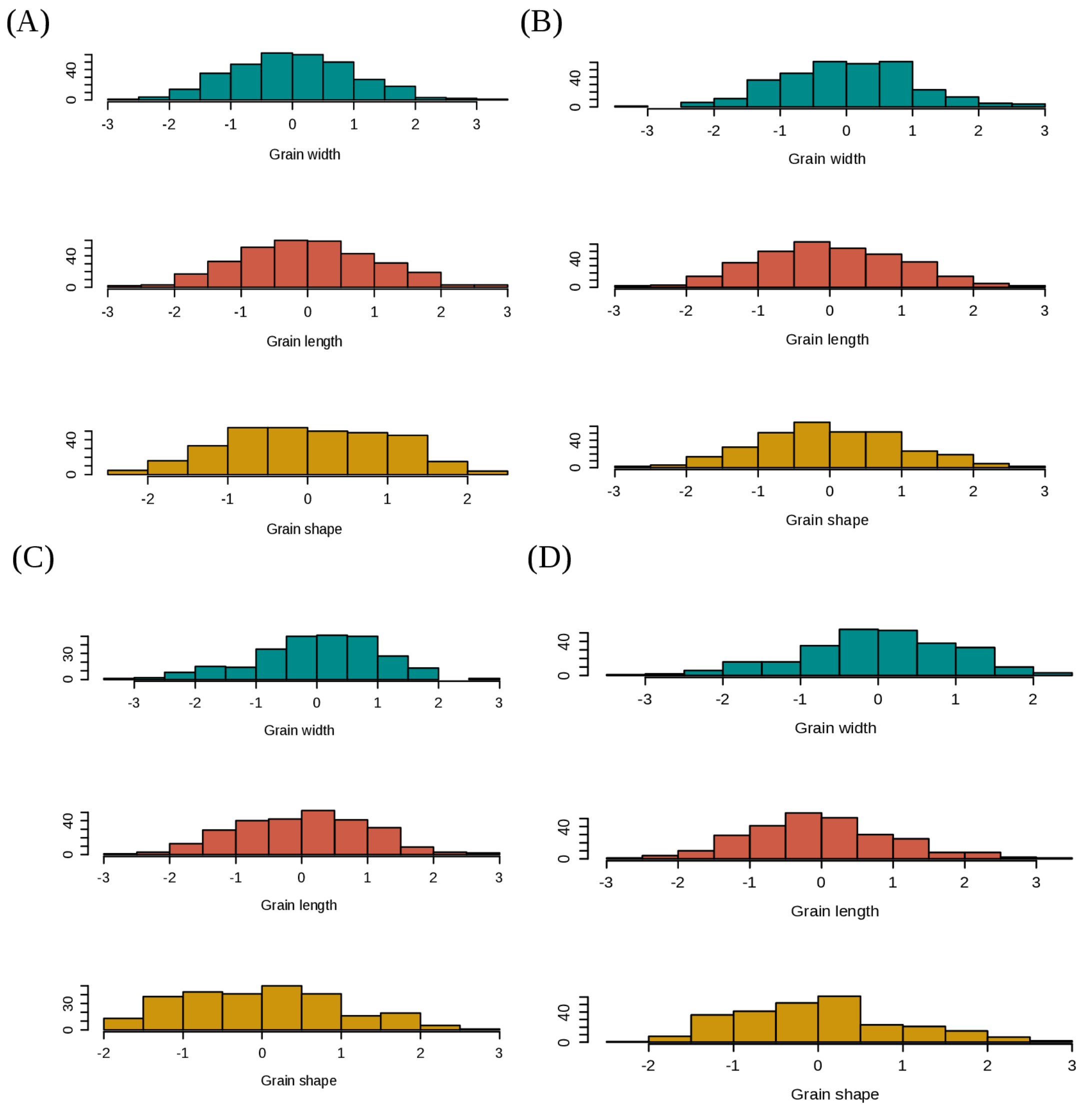

Figure S2: Transformed phenotypic variation for -raw and -cooked grain width, grain length and grain shape in indica and japonica using WarpedLMM. WarpedLMM uses the warping function where its search for most probable function instead of using a static function. Grain dimensions in indica in case of -raw (A) and -cooked grain (B); grain dimensions in japonica in -raw (C) and -cooked grain (D). The x-axis represents respective trait value after transformation and Y-axis represent number of samples.

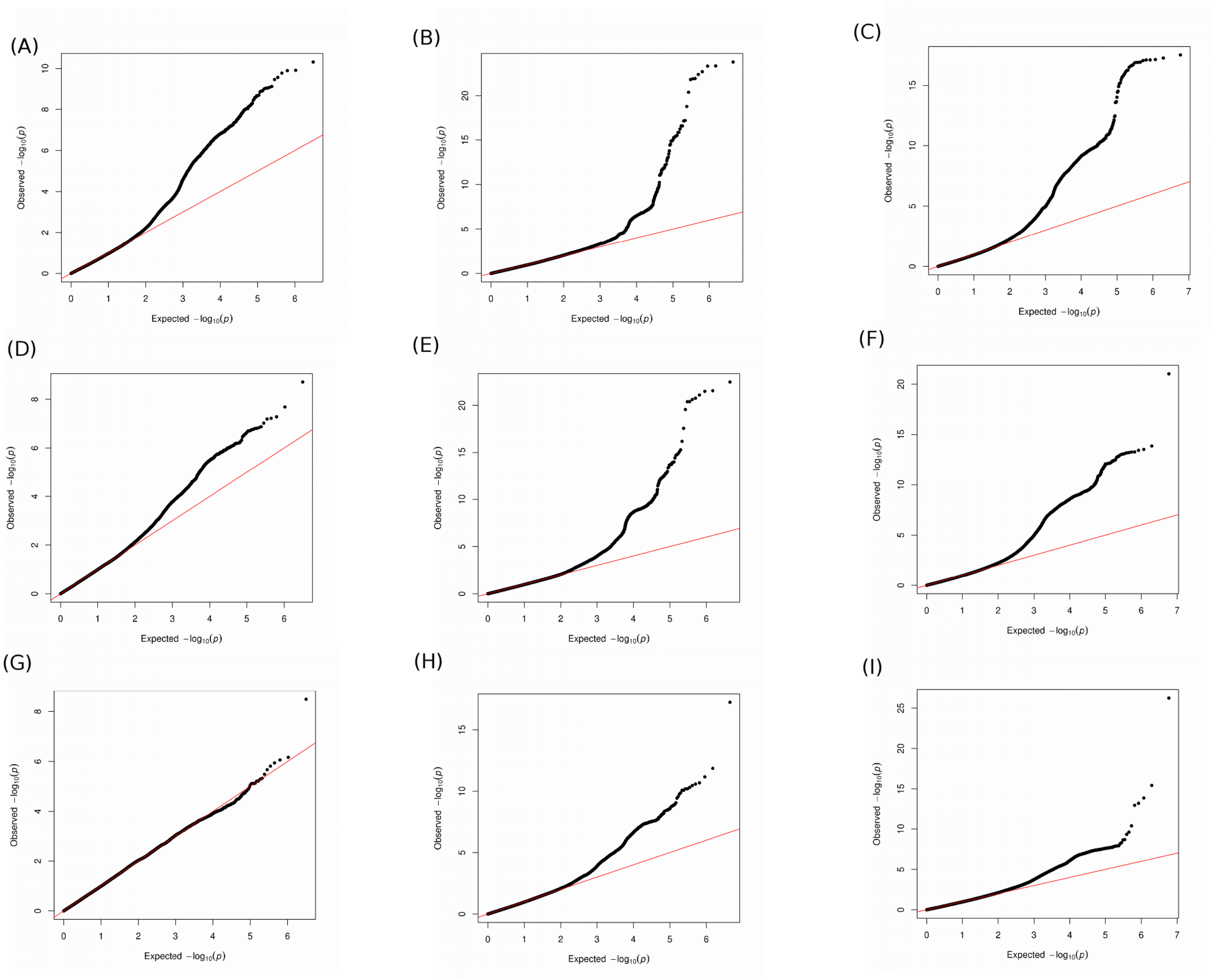

Figure S3: Q-Q plot for all the SNP-based single-locus GWAS study in -raw grain. Emmax was used for these association studies with covariates and kinship matrix as random effects. *Japonica* (top), *indica* (middle) and combined population (lower) are represented with vertically arranged grain width (A,D,G), grain shape (B,E,H) and grain length (C,F,I). Q-Q plots were created using qqman, an R package.

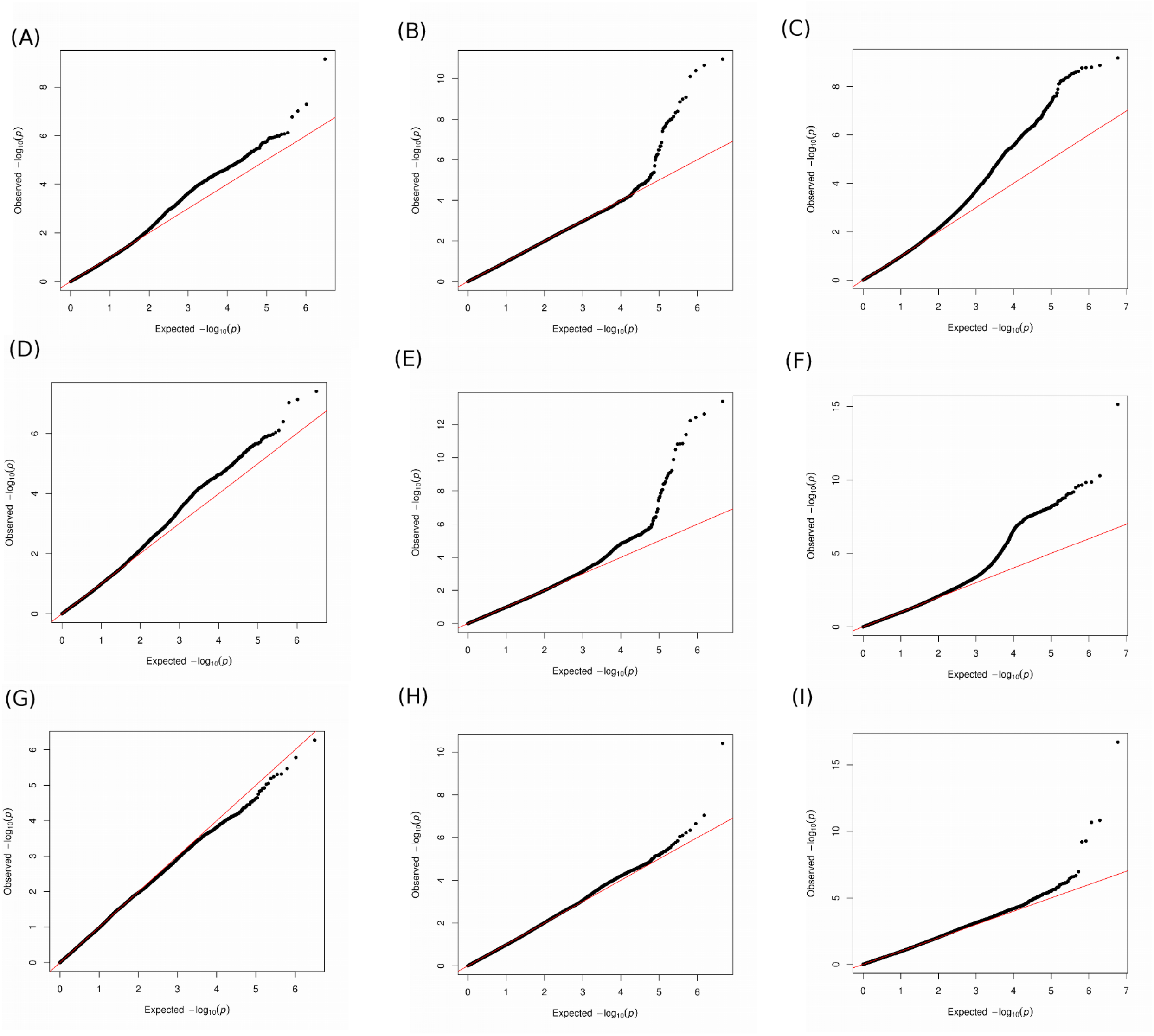

Figure S4: Q-Q plot for all the SNP-based single-locus GWAS study in -cooked grain. Emmax was used for these association studies with covariates and kinship matrix as random effects. *Japonica* (top), *indica* (middle) and combined population (lower) are represented with vertically arranged grain width (A,D,G), grain shape (B,E,H) and grain length (C,F,I). Q-Q plots were created using qqman, an R package.

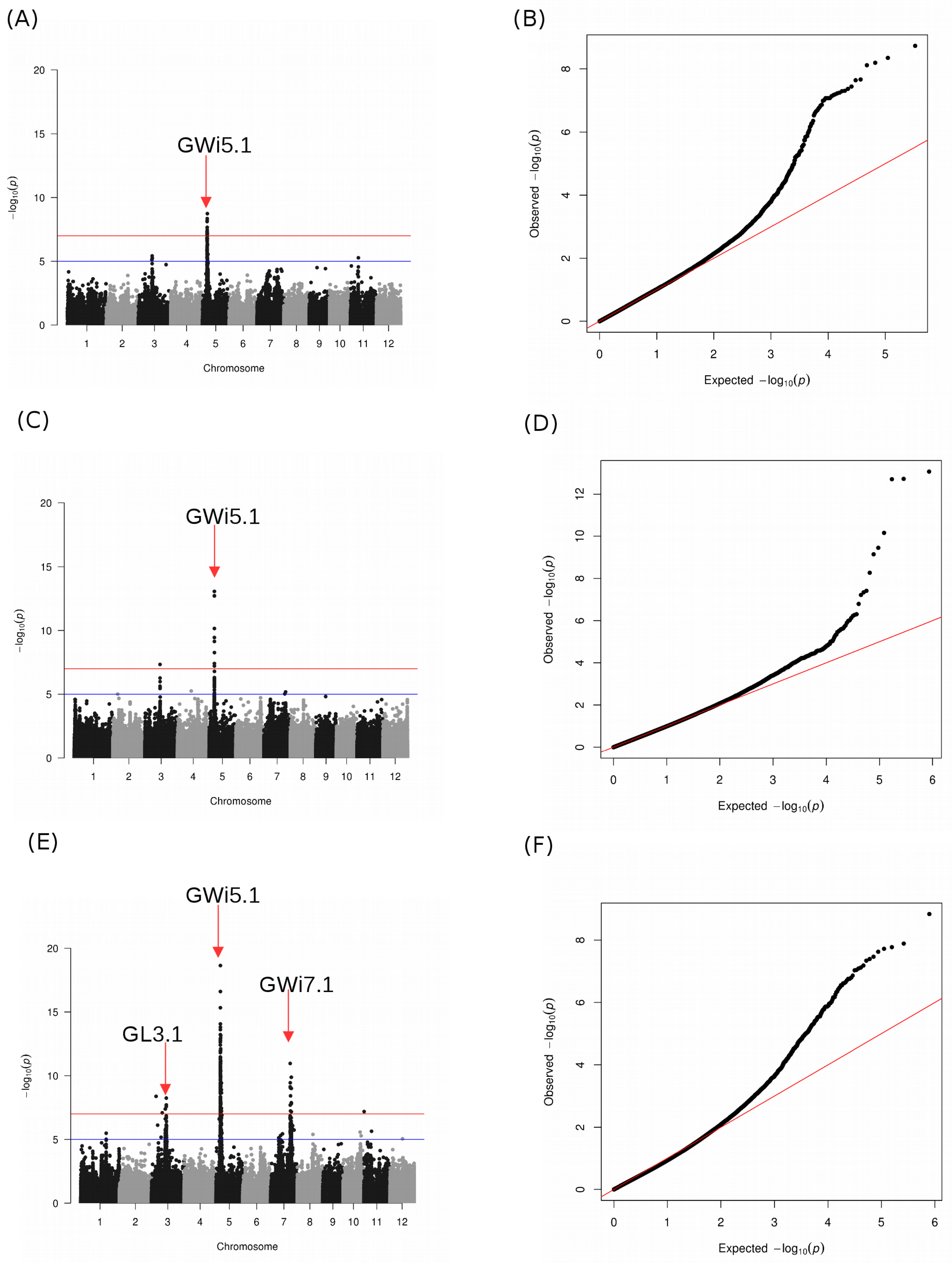
(A)
GWi5.1
−log10(p)
Chromosome
(B)
Observed −log10(p)
Expected −log10(p)
(C)
GWi5.1
−log10(p)
Chromosome
(D)
Observed −log10(p)
Expected −log10(p)
(E)
GWi5.1
GWi7.1
GL3.1
−log10(p)
Chromosome
(F)
Observed −log10(p)
Expected −log10(p)

Figure S5: Indel-based single-locus GWAS for raw grain width (GWi) trait and identification of the causal genomic loci on chromosome 3, 5, 7 contributing for GWi trait using EMMAX. Manhatton plots of the single-locus GWAS on GWi for *japonica* (A), *indica* (C) and combined (E) for -raw grain; Q-Q plot for *japonica* (B), *indica* (D) and combined (F) for the same on the right side. Horizontal red and blue lines represent genome wide and suggestive threshold, respectively. Manhatton and Q-Q plots were created by qqman, an R package. Emmax was used for the genome-wide association study with necessary covariates and kinship matrix.

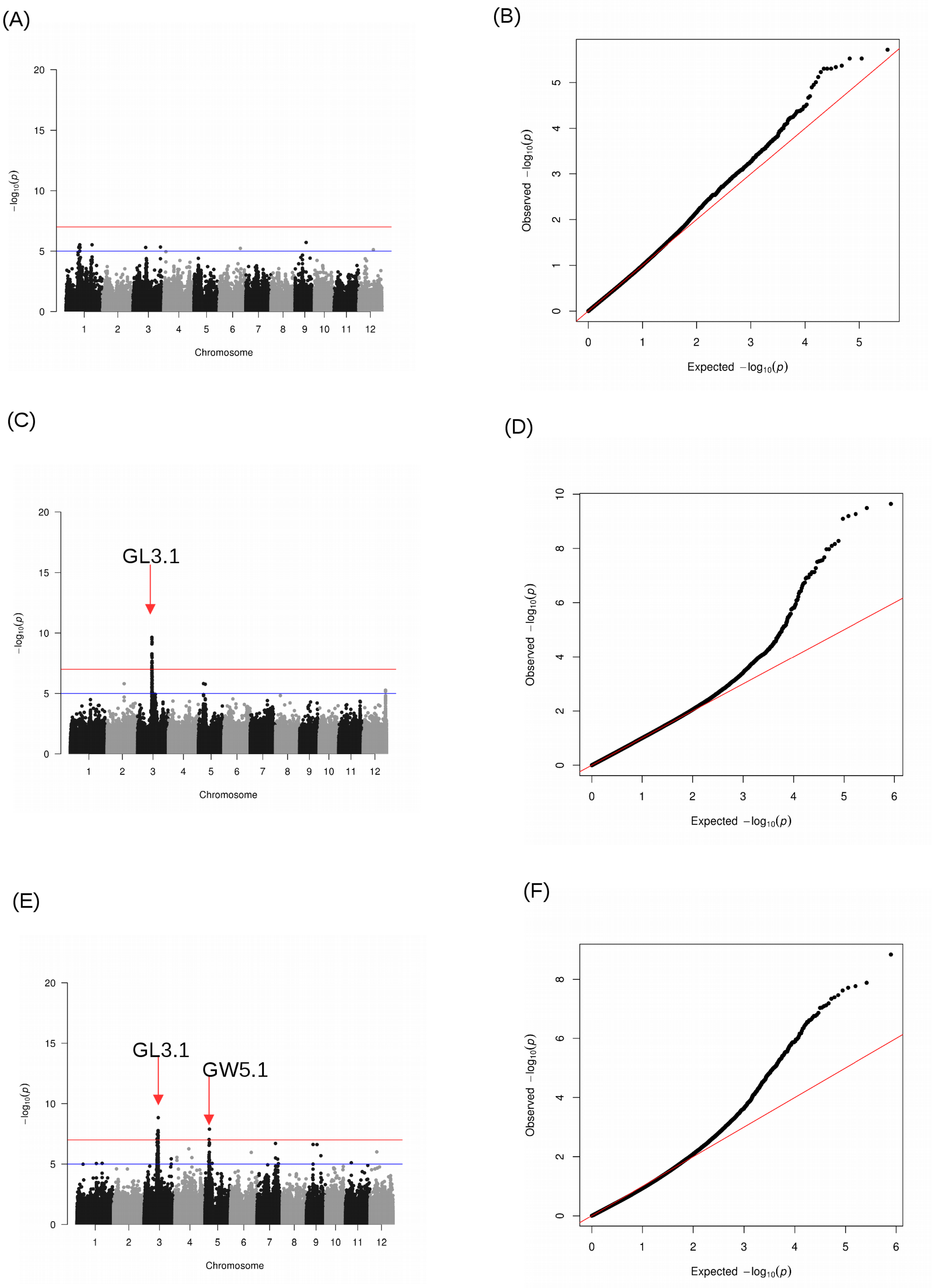

(A)
(B)
(C)
(D)
(E)
(F)
GL3.1
GW5.1
Chromosome
$-\log_{10}(p)$
Observed $-\log_{10}(p)$
Expected $-\log_{10}(p)$

Figure S6: Indel-based single-locus GWAS for raw grain length (GL) trait and identification of the causal genomic loci majorly on chromosome 3 using EMMAX. Manhatton plots of the single-locus GWAS on GL for *japonica* (A), *indica* (C) and combined (E) for -raw grain; Q-Q plot *japonica* (B), *Indica* (D) and combined (F) for the same on the right side. Horizontal red and blue lines represent genome wide and suggestive threshold, respectively. Red arrowhead indicates the GWAS peak.

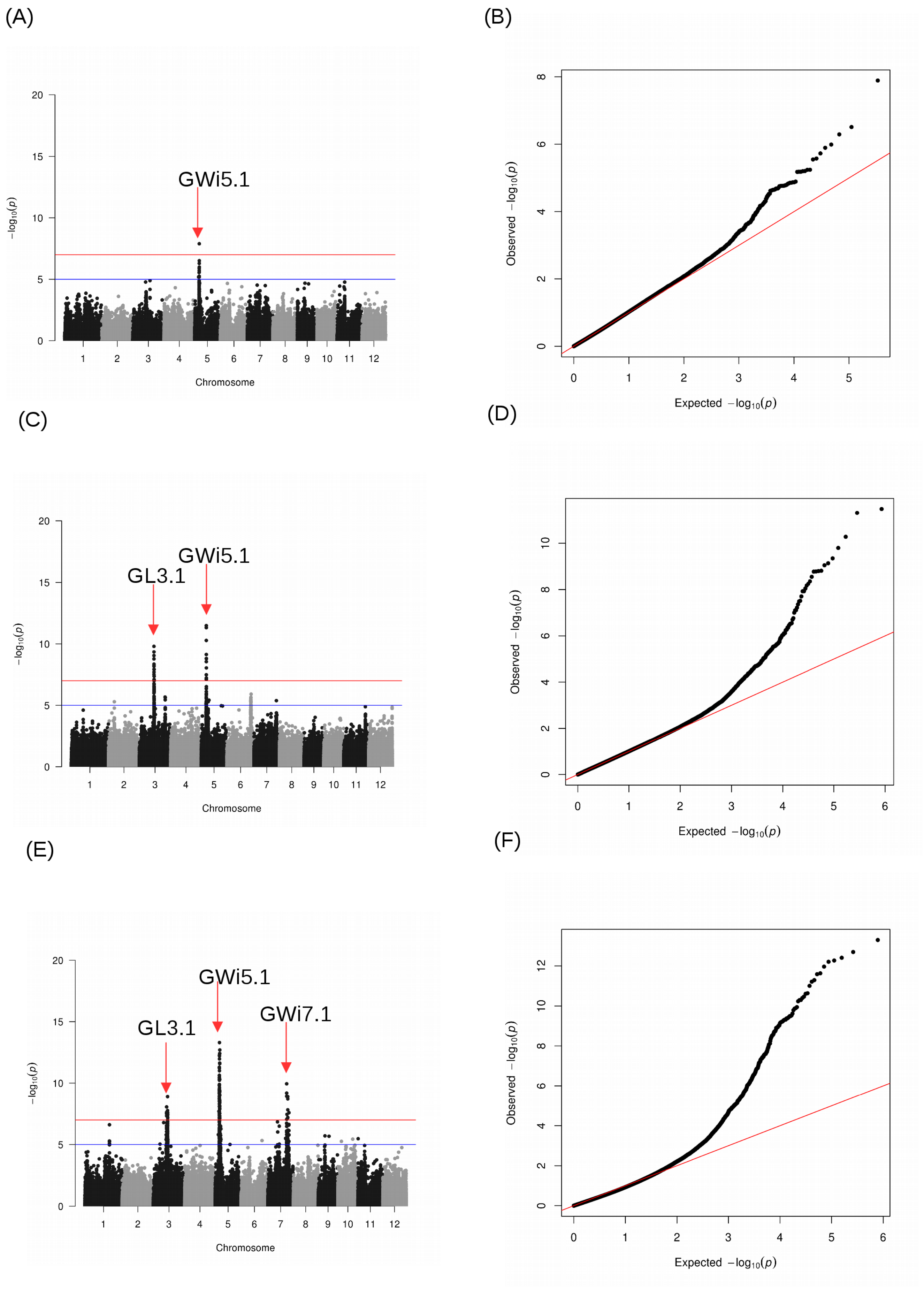
(A)
(B)
(C)
(D)
(E)
(F)
GWi5.1
GL3.1
GWi7.1
Chromosome
$-\log_{10}(p)$
Observed $-\log_{10}(p)$
Expected $-\log_{10}(p)$

Figure S7: Indel-based single-locus GWAS for raw grain shape (GS) trait and identification of the causal genomic region on chromosome 3, 5, 7 using EMMAX. Manhatton plots (left) and Q-Q plot (right) of the single-locus GWAS on GS for *japonica* (A,B), *indica* (C,D) and combined (E, F) panel. Horizontal red and blue lines represent genome-wide and suggestive threshold, respectively. The figures was created using qqman, an R package. Emmax was used to do the marker-trait association test with covariates and kinship matrix as random effects.

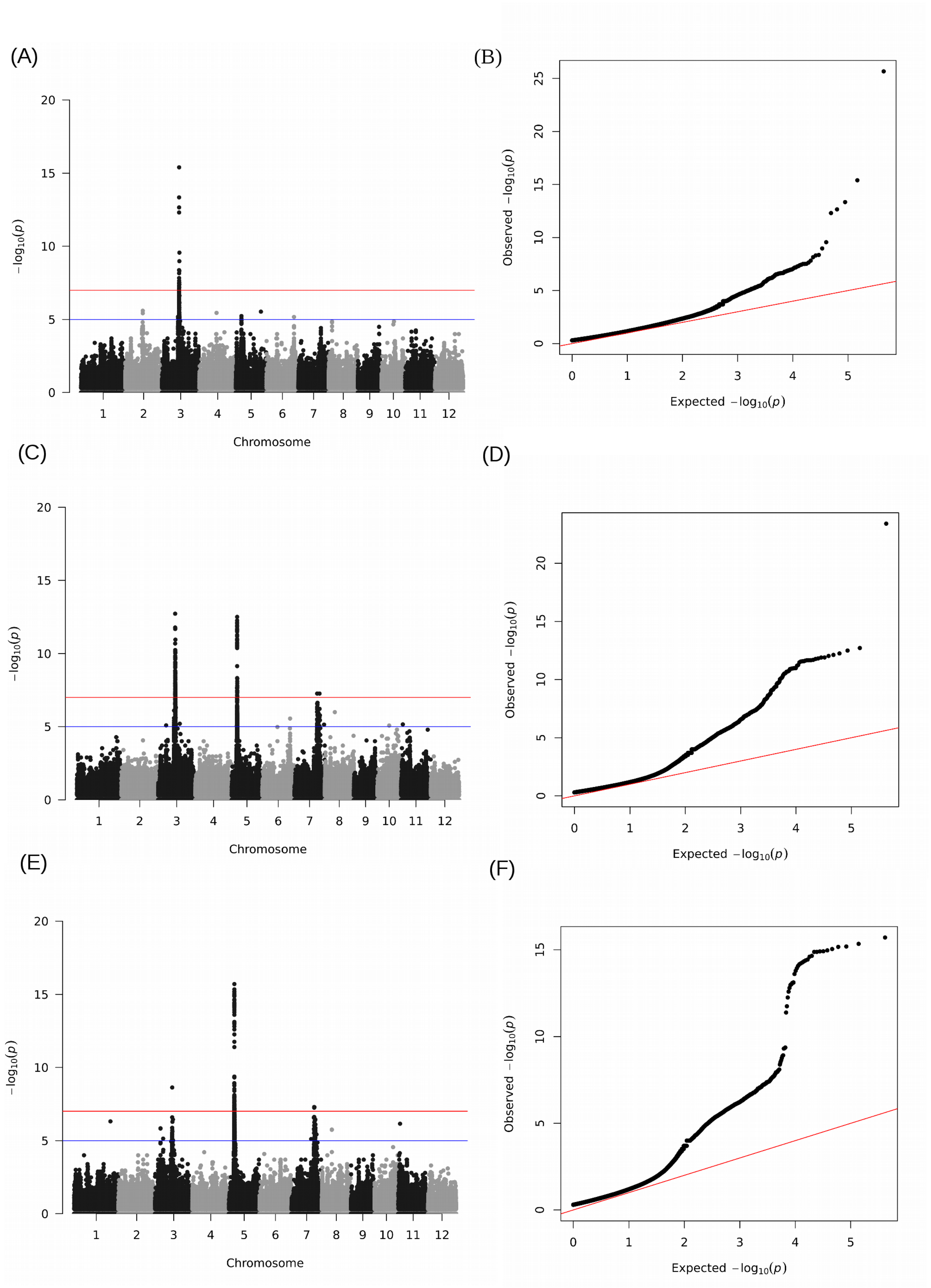

Figure S8: Representative Manhatton plot and Q-Q plot from SNP-based multi-locus association test using FASTmrEMMA. Manhatton plot for raw grain length (A), raw grain shape (C) and raw grain width (E) using combined population. Q-Q plot for raw grain length (B), raw grain shape (D) and raw grain width (F), using combined population. Red and blue lines in Manhatton plot are representing genome-wide and suggestive threshold, respectively. The plots were created by qqman, an R-package.

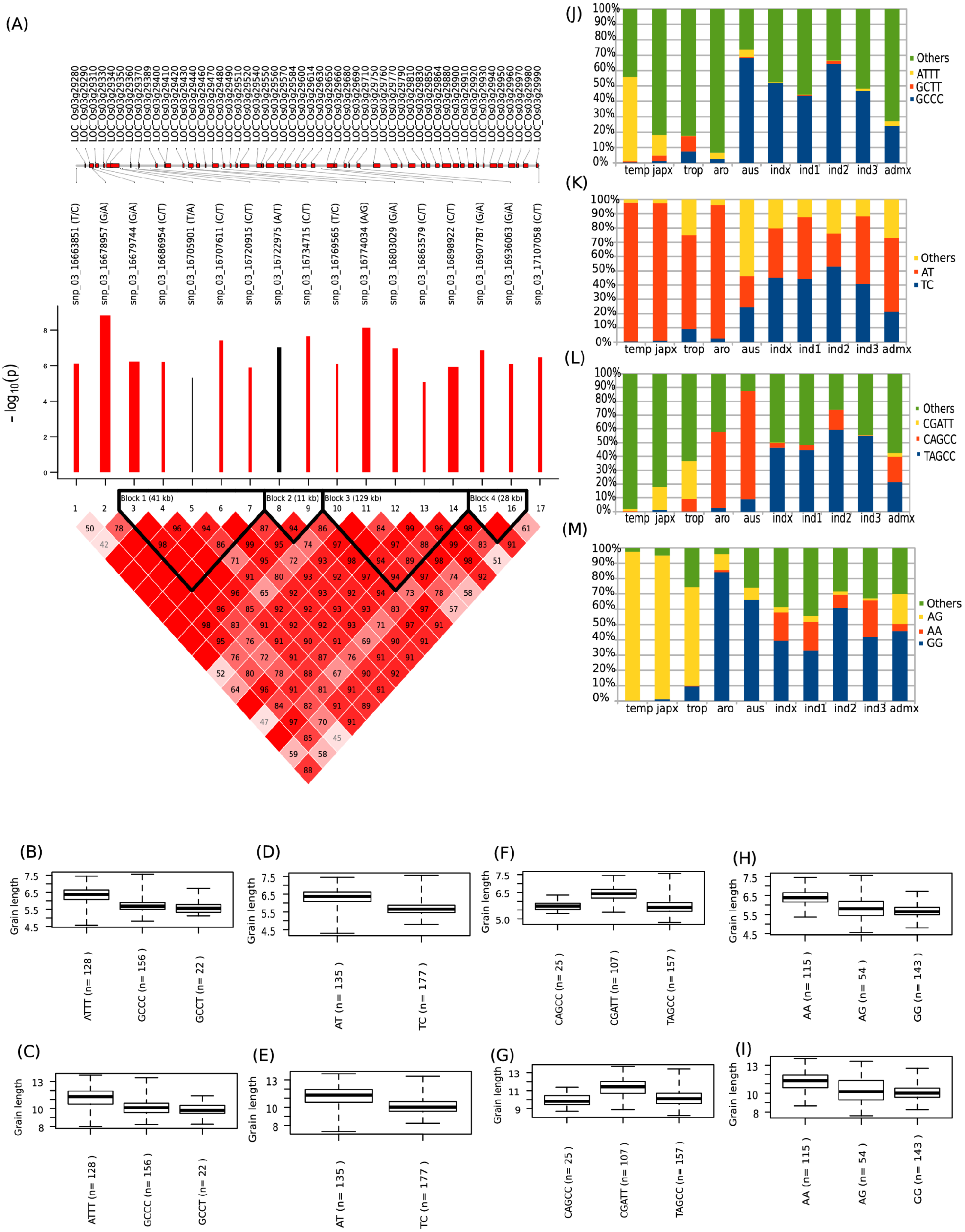
(A)
snp_03_16663851 (T/C)
snp_03_16678957 (G/A)
snp_03_16679744 (G/A)
snp_03_16686954 (C/T)
snp_03_16705901 (T/A)
snp_03_16707611 (C/T)
snp_03_16720915 (C/T)
snp_03_16722975 (A/T)
snp_03_16734715 (C/T)
snp_03_16769565 (T/C)
snp_03_16774034 (A/G)
snp_03_16803029 (G/A)
snp_03_16863579 (C/T)
snp_03_16898922 (C/T)
snp_03_16907787 (G/A)
snp_03_16936063 (G/A)
snp_03_17107058 (C/T)
- log10(p)
Block 1 (41 kb)
Block 2 (11 kb)
Block 3 (129 kb)
Block 4 (28 kb)
(B)
(C)
(D)
(E)
(F)
(G)
(H)
(I)
Grain length
ATTT (n= 128)
GCCC (n= 156)
GCCT (n= 22)
AT (n= 135)
TC (n= 177)
CAGCC (n= 25)
CGATT (n= 107)
TAGCC (n= 157)
AA (n= 115)
AG (n= 54)
GG (n= 143)
(J)
Others
ATTT
GCTT
GCCC
(K)
Others
AT
TC
(L)
Others
CGATT
CAGCC
TAGCC
(M)
Others
AG
AA
GG
temp japx trop aro aus indx ind1 ind2 ind3 admx

Figure S9: SNP-based GWAS for grain length trait and identification of genomic region on chromosome 3 in Indica sub-species significantly associated with grain length; (A) the linkage disequilibrium plot of the 17 tagged SNPs most significantly associated grain length. A scaled and highly dense plot of genomic region on the chromosome is shown where the positions of the genes are labeled with red boxes scaled according to in Nipponbare reference genome (MSU release version 7). The positions of the 17 tagged SNPs are indicated with the plot of significant p-values ($-\log_{10}(P)$) represented by red bars of respective SNPs where bar thickness indicate relative effect sizes (negative effect) of SNPs on grain length. Phenotype distribution of 4 blocks also shows as boxplot for -raw grain (B, D, F, H) and -cooked grain (C, E, G, I). The distribution of these blocks (J, K, L, M) was further examined with data from the 3000 Rice Genomes Project (3K-RGP).

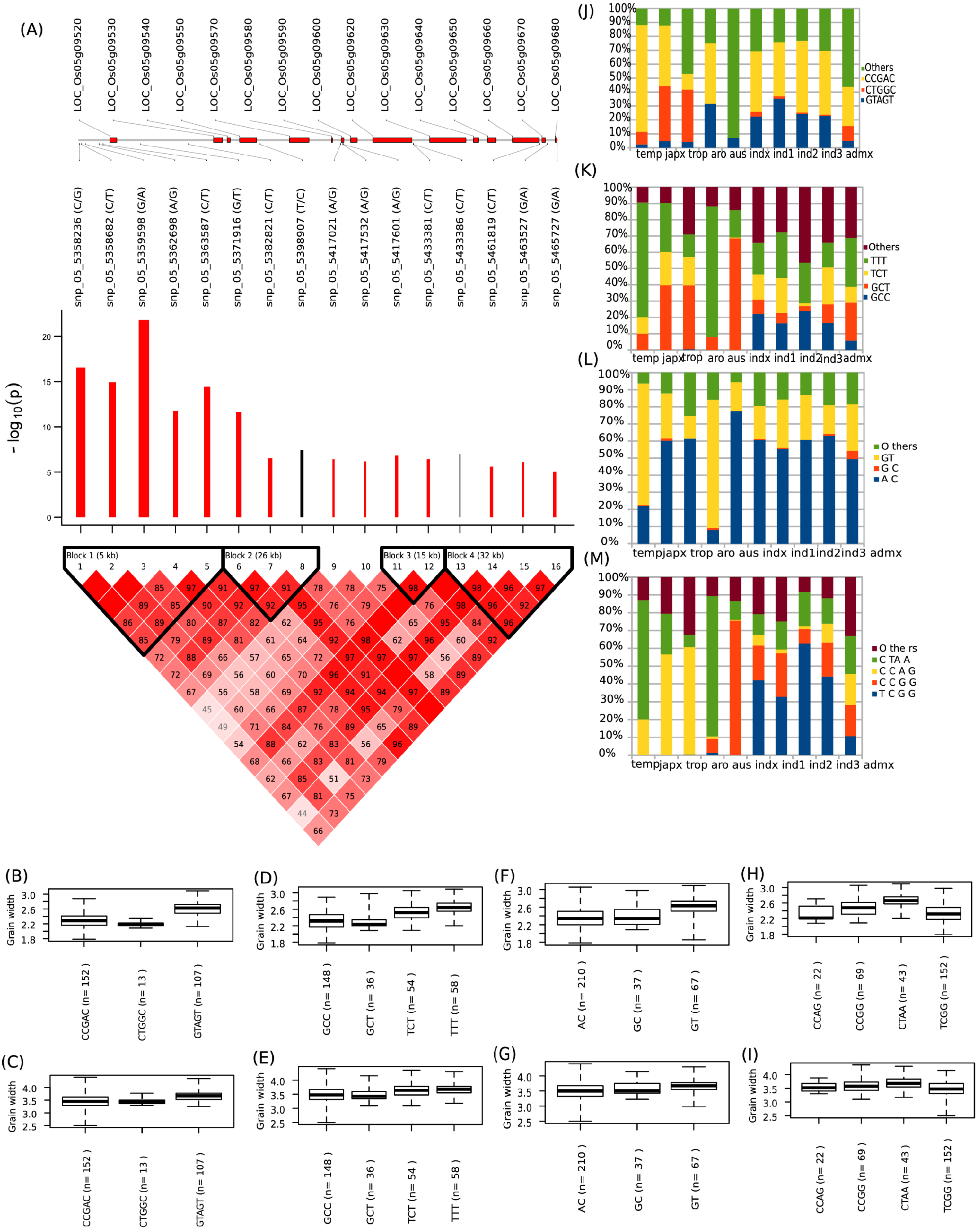
(A)
LOC_Os05g09520
LOC_Os05g09530
LOC_Os05g09540
LOC_Os05g09550
LOC_Os05g09570
LOC_Os05g09580
LOC_Os05g09590
LOC_Os05g09600
LOC_Os05g09620
LOC_Os05g09630
LOC_Os05g09640
LOC_Os05g09650
LOC_Os05g09660
LOC_Os05g09670
LOC_Os05g09680
snp_05_5358236 (C/G)
snp_05_5358682 (C/T)
snp_05_5359598 (G/A)
snp_05_5362698 (A/G)
snp_05_5363587 (C/T)
snp_05_5371916 (G/T)
snp_05_5382821 (C/T)
snp_05_5398907 (T/C)
snp_05_5417021 (A/G)
snp_05_5417532 (A/G)
snp_05_5417601 (A/G)
snp_05_5433381 (C/T)
snp_05_5433386 (C/T)
snp_05_5461819 (C/T)
snp_05_5463527 (G/A)
snp_05_5465727 (G/A)
- log10(p)
Block 1 (5 kb)
Block 2 (26 kb)
Block 3 (15 kb)
Block 4 (32 kb)
(B) (C) (D) (E) (F) (G) (H) (I)
Grain width
CCGAC (n= 152)
CTGGC (n= 13)
GTAGT (n= 107)
GCC (n= 148)
GCT (n= 36)
TCT (n= 54)
TTT (n= 58)
AC (n= 210)
GC (n= 37)
GT (n= 67)
CCAG (n= 22)
CCGG (n= 69)
CTAA (n= 43)
TCGG (n= 152)
(J)
Others
CCGAC
CTGGC
GTAGT
(K)
Others
TTT
TCT
GCT
GCC
(L)
O thers
GT
G C
A C
(M)
O the rs
C TA A
C C A G
C C G G
T C G G
temp japx trop aro aus indx ind1 ind2 ind3 admx

Figure S10: SNP-based GWAS for grain width trait and identification of genomic region on chromosome 5 in *indica* sub-species, significantly associated with grain width (Gwi); (A) the linkage disequilibrium plot of the 16 tagged SNPs most significantly associated grain width. A scaled and highly dense plot of genomic region on the chromosome is shown where the positions of the genes are represented as red boxes scaled according to in the Nipponbare reference genome (MSU release version 7). The positions of the 16 tagged SNPs are indicated with the plot of significant p-values ($-\log_{10}(P)$) represented by red bars of respective SNPs where bar thickness indicate relative effect sizes of SNPs on grain width; black bar implies positive additive effect, while red is the reverse); Four haplotype blocks identified from LD plot were represented with phenotype distribution as boxplot for -raw grain (B, D, F, H) and -cooked grain (C, E, G, I). Haplotypes formed from the 16 tagged SNPs depicted broad phenotypic variation for grain width. The distribution of these blocks (J, K, L, M) was further examined with data from the 3000 Rice Genomes Project (3K-RGP).

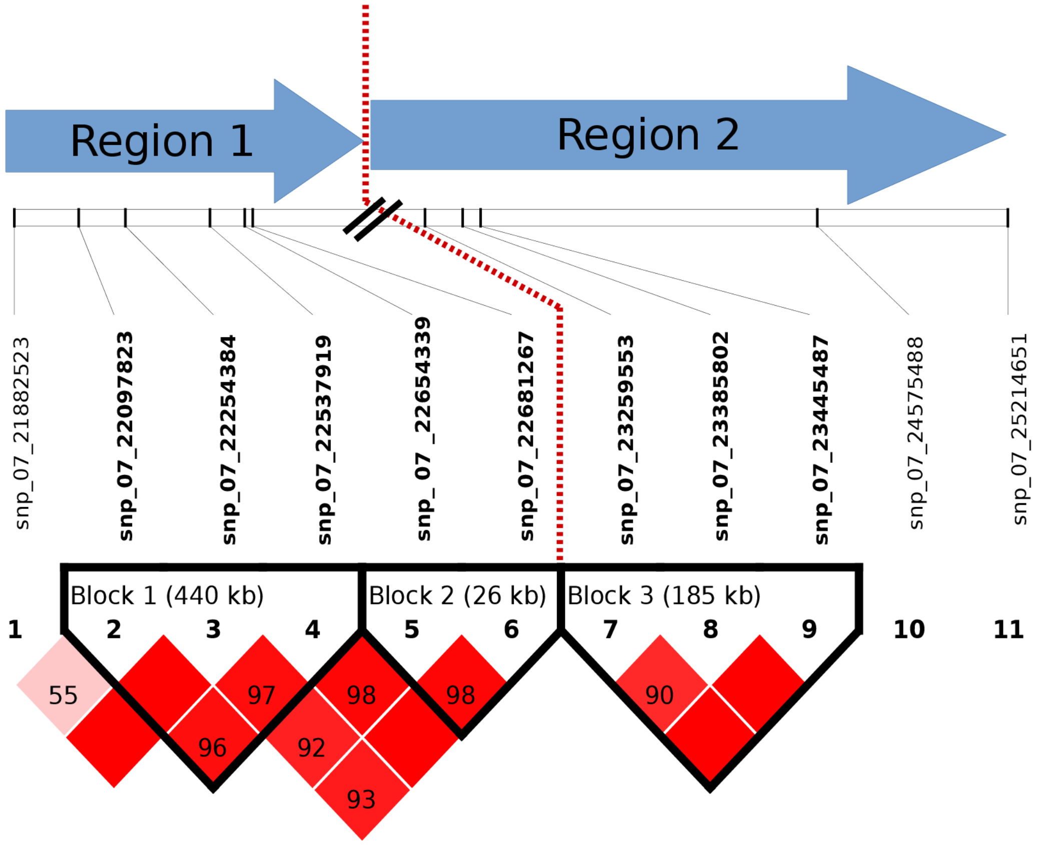

Figure S11: LD plots for SNP-based GWAS at chromosome 7, representing two genomic regions associated significantly with grain width trait. LD block 1 and 2 underlies in the region 1 whereas block 3 present in region 2. Region 1 (928kb) and 2 (1.9mb) is separated by ~550 kb from each other; red dotted line signifies the separation between both of the regions.

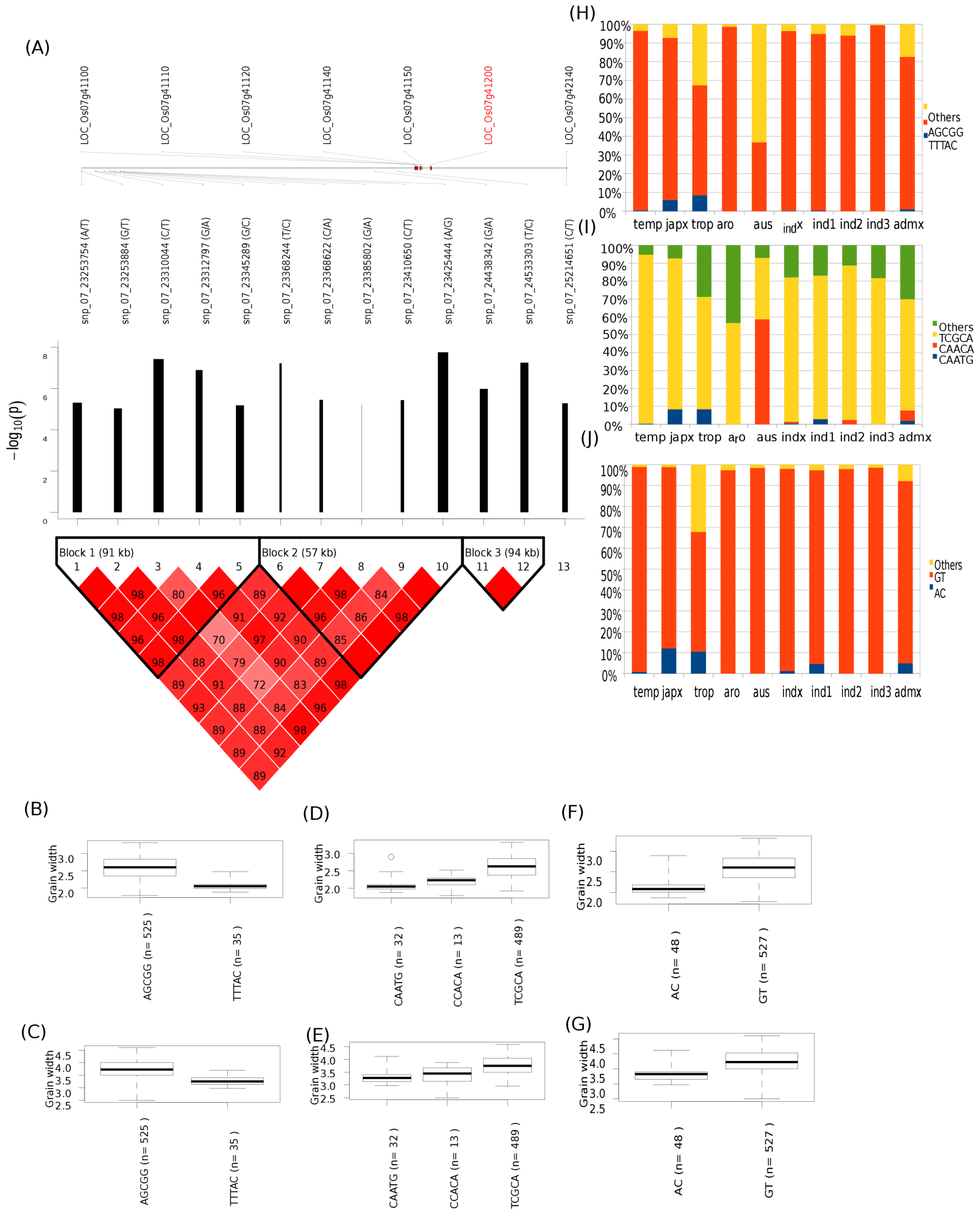

(A)
LOC_Os07g41100
LOC_Os07g41110
LOC_Os07g41120
LOC_Os07g41140
LOC_Os07g41150
LOC_Os07g41200
LOC_Os07g42140
snp_07_23253754 (A/T)
snp_07_23253884 (G/T)
snp_07_23310044 (C/T)
snp_07_23312797 (G/A)
snp_07_23345289 (G/C)
snp_07_23368244 (T/C)
snp_07_23368622 (C/A)
snp_07_23385802 (G/A)
snp_07_23410650 (C/T)
snp_07_23425444 (A/G)
snp_07_24438342 (G/A)
snp_07_24533303 (T/C)
snp_07_25214651 (C/T)
$-\log_{10}(p)$
Block 1 (91 kb)
Block 2 (57 kb)
Block 3 (94 kb)
(B)
Grain width
AGCGG (n= 525)
TTTAC (n= 35)
(C)
(D)
CAATG (n= 32)
CCACA (n= 13)
TCGCA (n= 489)
(E)
(F)
AC (n= 48)
GT (n= 527)
(G)
(H)
Others
AGCGG
TTTAC
temp japx trop aro aus indx ind1 ind2 ind3 admx
(I)
Others
TCGCA
CAACA
CAATG
(J)
Others
GT
AC

Figure S12: SNP-based GWAS for grain width trait and identification of genomic region 2 on chromosome 7 (GWi 7.2) in combined population significantly associated with grain width; (a) the linkage disequilibrium plot of the 13 tagged SNPs most significantly associated with grain width. A scaled and highly dense plot of genomic region on the chromosome is shown where the positions of the genes are labeled with red boxes scaled according to in the Nipponbare reference genome (MSU release version 7).

The positions of the 13 tagged SNPs are indicated with the plot of significant p-values ($-\log_{10}(P)$) represented by black bars of respective SNPs where bar thickness indicate relative effect sizes (positive effect) of SNPs on grain width; All the identified blocks were presented with phenotype as boxplot for -raw grain (B, D, F) and -cooked grain (C, E, G). The distribution 3k distribution of these blocks (H, I, J) is then examined with data from the 3000 Rice Genomes Project (3K-RGP).

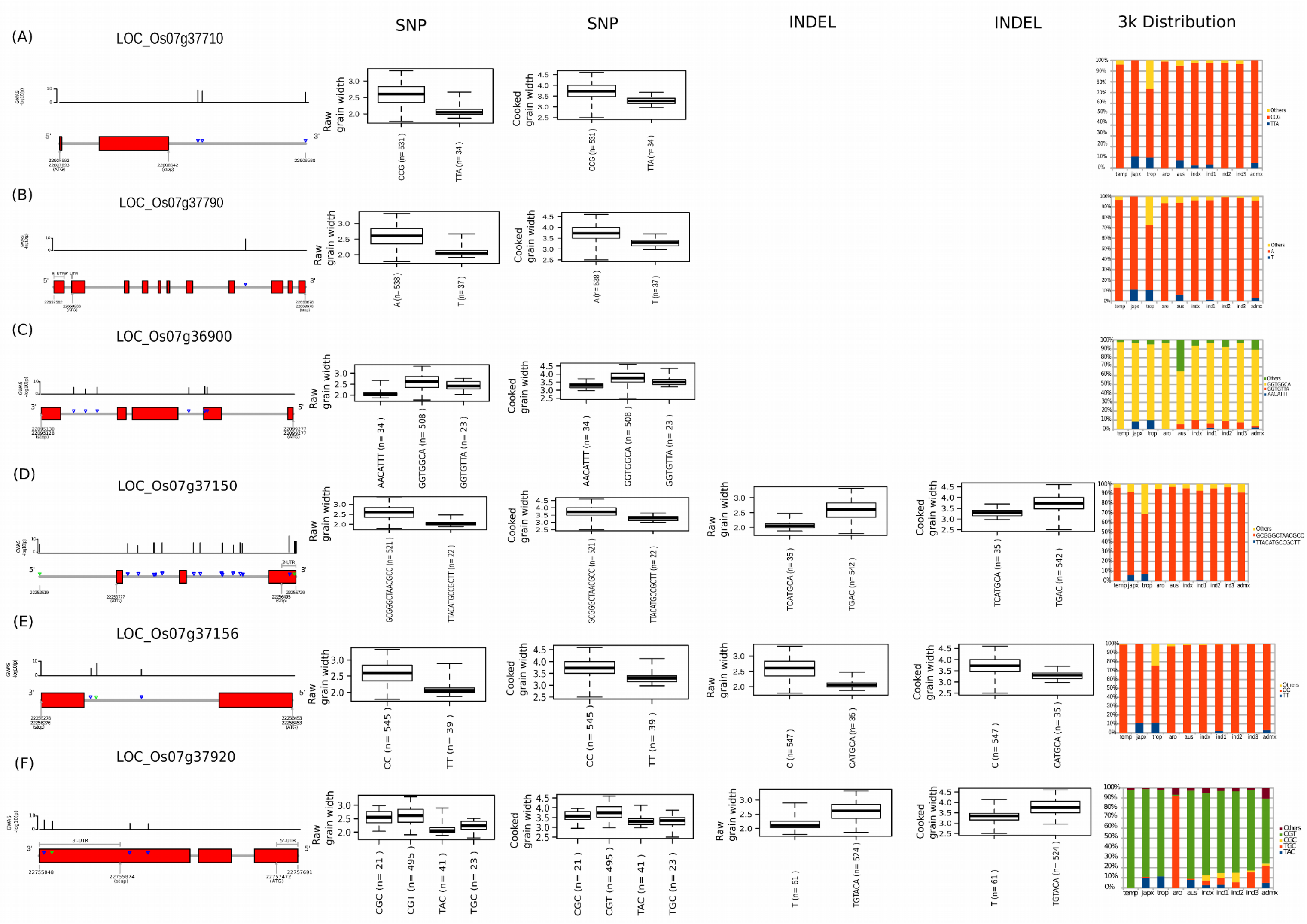

Figure S13: Targeted-gene association study (TGAS) in the region 1 of Chr7 (Gwi7.1). Gene structure along with phenotype distribution as boxplot and 3k distribution has represented for each gene. LOC_Os07g37710 (A); LOC_Os07g37790 (B); LOC_Os07g36900 (C); LOC_Os07g37150 (D); LOC_Os07g37156 (E); LOC_Os07g37920 (F).

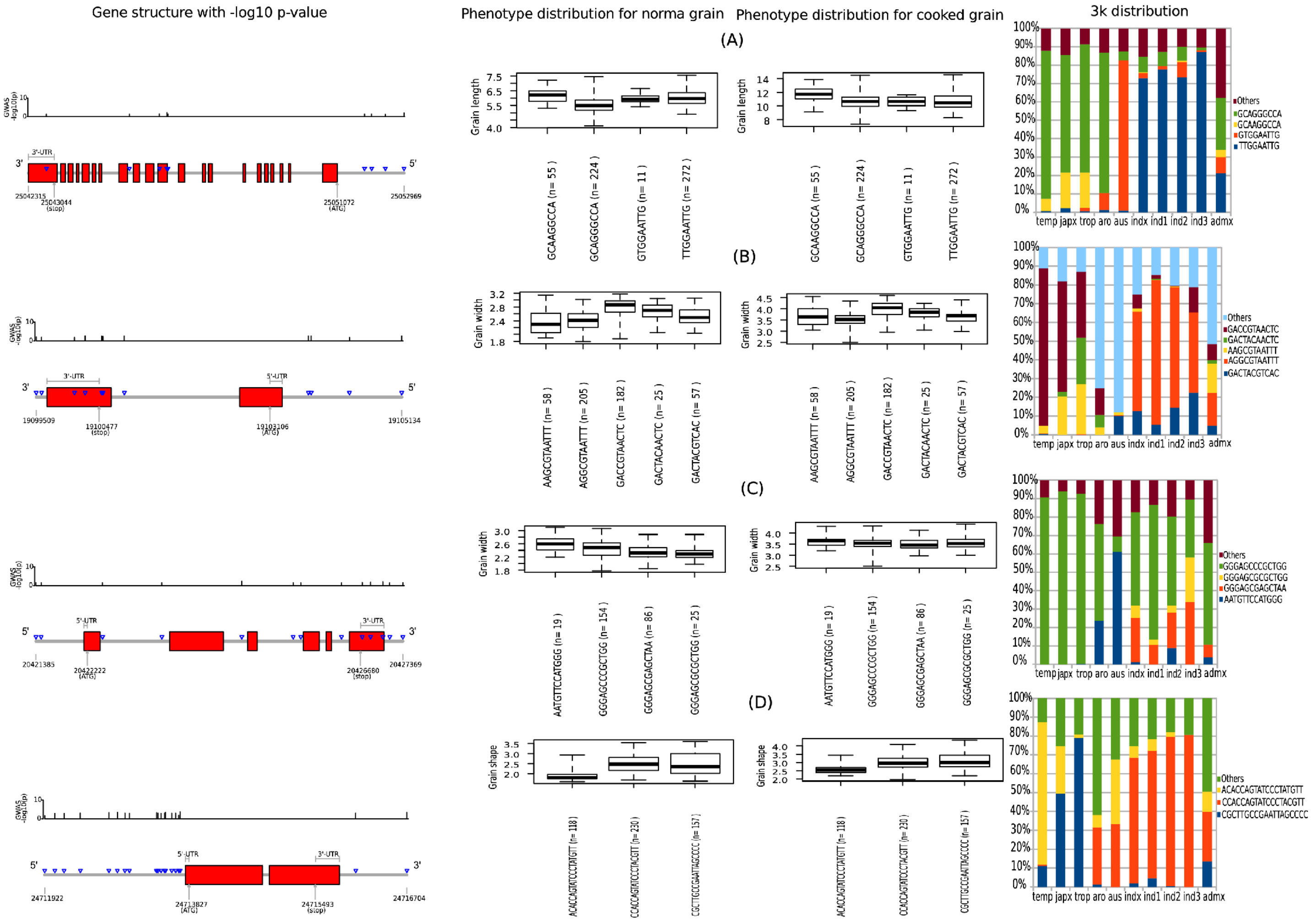

Figure S14: Gene structure with -log$_{10}$(P)-values, haplotype with phenotype distribution for -raw grain and -cooked grain and 3K-PGP distribution for some important cloned genes. (A) GL3.1; (B) GLW7; (C) GIF1; (D) big-grain-2.

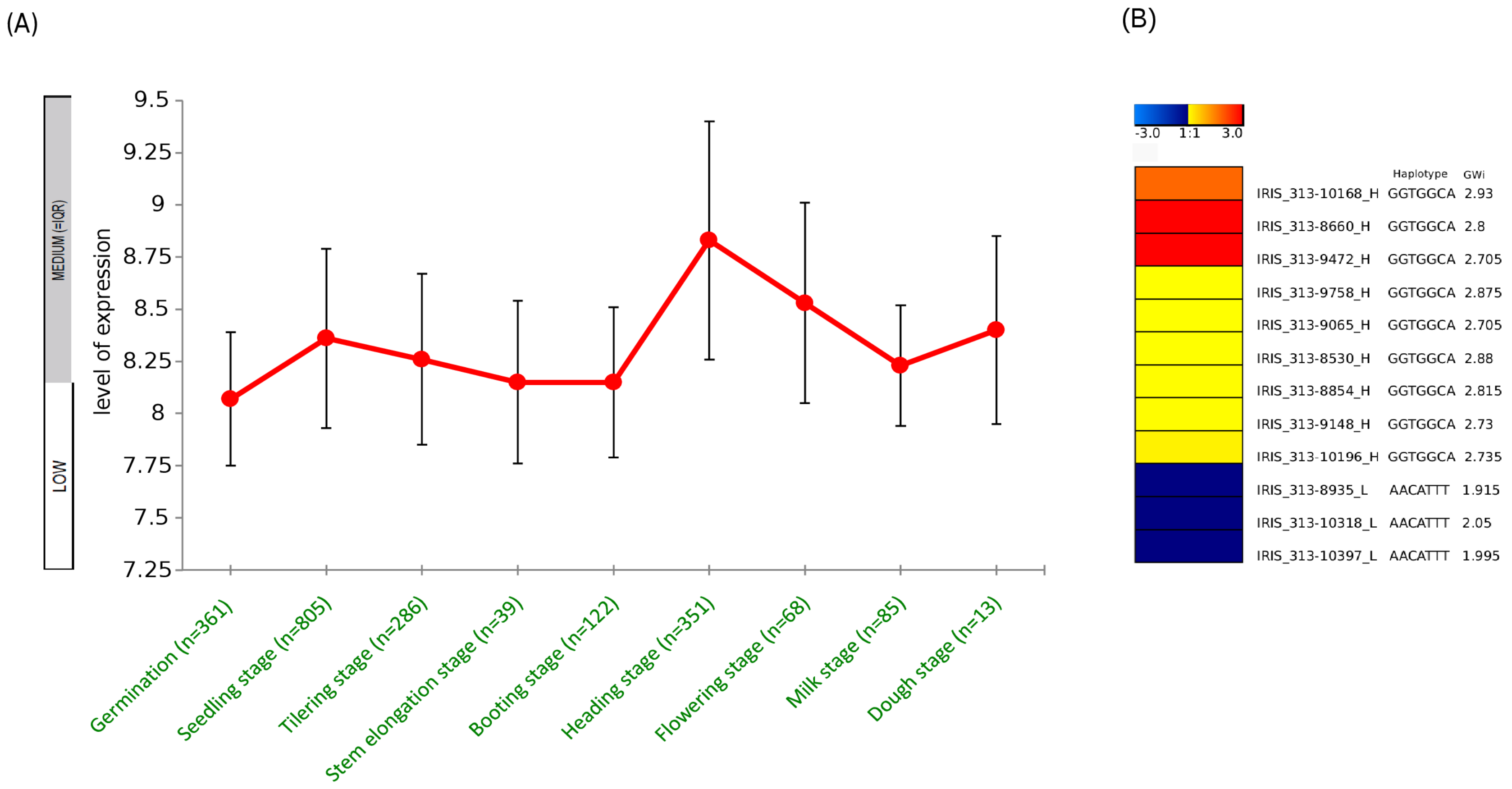

Figure S15: Representative expression profiling of GWi-associated gene LOC_Os07g36900, (A) Level of expression of gene LOC_Os07g36900 determined by using Affymetrix rice genome array at Genevestigator platform (https://genevestigator.com/gv/), across different developmental stages. The log2 transform values of signal intensity appeared on Affymetrix rice genome array were used to construct a plot. The medium level of expression was estimated in later stages of development with relatively higher expression in heading stage, whereas, low to medium level of expression observed during early developmental stages. Here, X-axis represented the specific plant developmental stage, whereas, y-axis represented the level of expression signal that signifies the signal intensity on Affymetrix rice genome array, (B) differential expression profile of gene LOC_Os07g36900 in genotypes with high and low grain width mentioned as a suffix (-H) and (-L), respectively along with the accession name. The contributing haplotype and the grain width (GWi) phenotype of the respective line were also represented on the right-hand side of the expression map.

Supplementary Table S4: List of cloned and characterized genes for grain size/shape in rice and detected causal variation through TGAS.

Gene/QTL	Locus IDs	Encoded protein	Trait Regulation (*reference*)*	Syntenic description	Position	SNP variation		Trait affected (population)#	*β-value*	*P-value*
						Ref	Alt			
Big grain 1	LOC_Os03g07920	Novel membrane-localized protein likely to involve in auxin transport	Positively regulate grain length[1]	Syntenic	4039146	A	G	GL(C)	0.1736	4.85E-02
An-1	LOC_Os04g28280	Basic Helix-Loop-Helix Protein	Positively regulate grain length[2]	Syntenic falling in break point region	16732643	T	G	GL(J)	-0.2227	1.63E-03
GS3	Os03g0407400 (RAP-db ID) Corresponding ID not present in MSU version 7	A putative G protein γ subunit	Negatively regulates grain size[3,4]	Syntenic Decay in collinearity block	16733441<br>16734618<br>16734121<br>16734333	C<br>G<br>G<br>T	A<br>A<br>T<br>G	GL(C)<br>GL(I)<br>GL(I)<br>GL(I)	-0.4975<br>-0.3715<br>-0.3601<br>-0.3662	5.83E-27<br>7.15E-09<br>1.22E-08<br>1.99E-08
qGL3/GL3.1	LOC_Os03g44500	Protein phosphatase with Kelch-like repeat domain	Limiting cell proliferation[5-7]	Syntenic Decay in collinearity block	25052969<br>25046243<br>25046030	A<br>A A	G<br>G<br>G	GS(C)<br>GL(C)<br>GS (I)	0.2109<br>-0.1551<br>0.2139	2.77E-03<br>4.88E-03<br>9.09E-03
GW2	LOC_Os02g14720	RING-type E3 ubiquitin ligase	Restricts cell proliferation in spikelet hulls[8]	Syntenic	8122148<br>8114649	T<br>A	C<br>T	GWi(I)<br>GWi(J)	0.1419<br>-0.1508	8.83E-03<br>3.18E-02
SEED WIDTH ON CHROMOS OME 5 (qSW5/GW5)	LOC_Os05g09510-LOC_Os05g09520* (functional polymorphism between both genes)	A ubiquitin-related protein	Grain width by limiting cell proliferation[9,10]	Syntenic Falling in break point region	5361329<br>5361329<br>5361276<br>5361276<br>5361509<br>5361509<br>5362756	A<br>A<br>T<br>T<br>C<br>C<br>T	G<br>G<br>C<br>C<br>A<br>A<br>C	GS (C)<br>GL (C)<br>GS (I)<br>GWi (I)<br>GWi (J)<br>GL (J)<br>Gwi (C)	-0.3324<br>-0.2389<br>0.4585<br>-0.5137<br>0.4520<br>-0.2457<br>0.3774	1.03E-07<br>1.56E-03<br>2.48E-19<br>2.38E-21<br>5.05E-09<br>1.56E-03<br>6.27E-09
GW8/OsSPL 16	LOC_Os08g41940	An protein with unknown function	Promotes cell division and grain filling[11]	Syntenic Falling in neighborhood break point region	26503280	A	T	GL (C)	-0.1188	2.99E-02
GS5	LOC_Os05g06660	A putative serine carboxypeptidase	Promotes cell proliferation in spikelet hulls[12]	Syntenic	3439806<br>3444708	T<br>G	G<br>A	GS (C)<br>GL (J)	0.1395<br>-0.3585	3.83E-03<br>1.03E-02
GL2	LOC_Os02g47280	Growth-Regulating Factor 4 (OsGRF4)	Both cell expansion and cell proliferation[13,14]	Syntenic	28862688	T	C	Gwi (C)	0.1070	4.29E-02
GL7/GW7/S	LOC_Os07g41200	Protein	Increasing cell	Syntenic	24665290	A	G	Gwi (C)	0.1585	5.52E-03

LG7		homologous to Arabidopsis thaliana LONGIFOLIA1/2	elongation in spikelet hulls[15-17]		24665290	A	G	GS (C)	-0.1852	5.52E-04
					24669663	C	G	GS (I)	-0.2763	1.95E-02
					24666398	G	A	GS (J)	0.1630	1.75E-02
GLW7	LOC_Os07g32170	Transcription factor OsSPL13	Positively regulates cell size in the grain hull[18]	Syntenic	19100263	T	C	GS (I)	0.1804	1.50E-03
					19100865	G	A	GWi (I)	-0.2254	2.46E-04
					19103121	G	C	GL (C)	0.1537	2.34E-02
					19105134	T	C	GS (C)	-0.1149	1.93E-03
					19100263	T	C	GWi (C)	-0.1299	2.19E-03
SRS3	LOC_Os05g06280	An unknown protein with kinesin motor domain and coiled coil structure	Positively regulate grain length[19]	Syntenic	3202088	C	A	GS (I)	-0.1506	5.75E-03
					3210838	C	A	Gwi (I)	0.1425	1.54E-02
					3210838	A	C	GS (C)	0.1138	2.11E-02
					3202088	A	C	GL (B)	0.1174	3.67E-02
SRS5	LOC_Os11g14220	An alpha-tubulin protein	Positively regulate grain length by cell elongation[20]	Syntenic Falling in neighborhood break point region	7958592	A	T	GS (I)	0.2834	5.55E-03
					7958592	A	T	Gwi (I`)	-0.2475	2.27E-02
Small grain 1 (smg1)	LOC_Os02g54600	A mitogen-activated protein kinase kinase 4	Effect grain size by cell proliferation[21]	Syntenic	33445344	A	G	GL (C)	-0.1543	2.10E-02
					33441336	C	G	GWi (I)	-0.2335	3.43E-02
THOUSAND -GRAIN WEIGHT 6 (TGW6)	LOC_Os06g41850	A novel protein with indole-3-acetic acid (IAA)-glucose hydrolase activity	Effect grain size by controlling grain filling[22]	Syntenic Falling in neighborhood break point region	25094756	A	G	GWi (C)	-0.1460	3.59E-02
SHORT GRAIN1	LOC_Os09g28520	A protein with unknown function	Negatively regulate grain size by decreasing Organ Elongation[23]	Syntenic	17353421	C	A	Gwi (C)	0.3895	8.83E-03
					17353421	C	A	GS (C)	-0.2859	3.99E-02
					17353176	T	C	GS (J)	-0.1682	4.91E-02
HGW	LOC_Os06g06530	Ubiquitin-Associated (UBA) domain protein	Negatively regulate grain size[24]	Syntenic	Not detected					
GIF1 (GRAIN INCOMPLETE FILLING 1)	LOC_Os04g33740	A cell-wall invertase required for grain filling	Positively regulate grain size[25]	Syntenic	204245682	A	G	GS (J)	0.2144	7.75E-03
					0424568	A	G	GL (C)	0.2152	7.69E-03
					20424568	A	G	GS (C)	0.2095	1.94E-03
					20426957	C	T	GWi (C)	0.2743	3.28E-02
					20424744	T	G	GS (I)	0.2673	3.55E-03
Flo2	LOC_Os04g55230	Encode protein with tetratricopeptide repeat motif to	Positively regulate grain size[26]	Syntenic	Not detected					

		mediate a protein–protein interactions								
SRS1/DEP2	LOC_Os07g42410	A novel protein of 1353 amino acid residues with unknown functional domains	Positively regulate grain length[27]	Syntenic	25381724<br>25381724	T<br>T	A<br>A	GL (C)<br>GL (J)	-0.1406<br>-0.1424	1.46E-02<br>2.15E-02
Dwarf11/ CPB1	LOC_Os04g39430	Cytochrome P450 (CYP724B1) enzyme	Positively regulate grain length[28]	Syntenic	23471496	C	G	GWi (I)	0.1343	2.21E-02
Big grain 2	LOC_Os07g41240	A cytochrome P450 Enzyme	Positively regulate grain size[29]	Syntenic	24713710<br>24713710<br>24712882<br>24712882<br>24713710<br>24712882	C<br>C<br>A<br>G<br>C<br>A	G<br>G<br>G<br>A<br>G<br>G	GWi (I)<br>GS (I)<br>GWi (J)<br>GL (C)<br>GS (C)<br>GS (J)	0.3041<br>-0.2915<br>-0.1500<br>-0.1857<br>-0.1951<br>0.1541	9.51E-03<br>9.13E-03<br>3.90E-02<br>3.50E-03<br>2.05E-04<br>1.64E-02
Smg11/D2	LOC_Os01g10040	A cytochrome P450 Enzyme	Positively regulate grain size[30]	Syntenic	5235440<br>5240482	C<br>A	A<br>G	GL (C)<br>GL (J)	-0.1246<br>0.2009	4.63E-02<br>4.00E-02
GS6/D62	LOC_Os06g03710	A GRAS-domain protein	Negatively regulate grain size[31]	Syntenic	1467292<br>1470261<br>1467292	T<br>T<br>T	G<br>C<br>G	GL (C)<br>GS (I)<br>GL (J)	0.1594<br>0.1301<br>0.1445	5.91E-03<br>3.84E-02<br>2.56E-02

*Mentions the type of gene regulation and reference of the study (mentioned as superscript) in the respective gene/QTL.
#letter mentioned in parenthesis depicts the SNPs detected in combined (ALL) germplasm panel (C), in only *Japonica* (J) and *indica* (I)

<u>**References**</u>

1 Liu, L. *et al.* Activation of Big Grain1 significantly improves grain size by regulating auxin transport in rice. *Proceedings of the National Academy of Sciences* **112**, 11102-11107 (2015).

2 Luo, J. *et al.* An-1 encodes a basic helix-loop-helix protein that regulates awn development, grain size, and grain number in rice. *The Plant Cell* **25**, 3360-3376, doi:10.1105/tpc.113.113589 (2013).

3 Fan, C. *et al. GS3*, a major QTL for grain length and weight and minor QTL for grain width and thickness in rice, encodes a putative transmembrane protein. *Theor. Appl. Genet.* **112**, 1164–1171, doi:10.1007/s00122-006-0218-1 (2006).

4 Mao, H. *et al.* Linking differential domain functions of the GS3 protein to natural variation of grain size in rice. *Proc. Natl. Acad. Sci. U. S. A.* **107**, 19579–19584, doi:10.1073/pnas.1014419107 (2010).

5 Hu, Z. *et al.* A Kelch motif-containing serine/threonine protein phosphatase determines the large grain QTL trait in rice. *J. Integr. Plant Biol.* **54**, 979-990 (2012).

6 Qi, P. *et al.* The novel quantitative trait locus GL3. 1 controls rice grain size and yield by regulating Cyclin-T1; 3. *Cell Res.* **22**, 1666-1680 (2012).

7 Zhang, X. *et al.* Rare allele of OsPPKL1 associated with grain length causes extra-large grain and a significant yield increase in rice. *Proceedings of the National Academy of Sciences* **109**, 21534-21539 (2012).

8 Song, X.-J., Huang, W., Shi, M., Zhu, M.-Z. & Lin, H.-X. A QTL for rice grain width and weight encodes a previously unknown RING-type E3 ubiquitin ligase. *Nature Genet.* **39**, 623–630 (2007).

9 Shomura, A. *et al.* Deletion in a gene associated with grain size increased yields during rice domestication. *Nature Genet.* **40**, 1023-1028, doi:10.1038/ng.169 (2008).

10 Weng, J. *et al.* Isolation and initial characterization of GW5, a major QTL associated with rice grain width and weight. *Cell Res.* **18**, 1199-1209, doi:10.1038/cr.2008.307 (2008).

11 Wang, S. *et al.* Control of grain size, shape, and quality by *OsSPL16* in rice. *Nature Genet.* **44**, 950–954, doi:10.1038/ng.2327 (2012).

12 Li, Y. *et al.* Natural variation in GS5 plays an important role in regulating grain size and yield in rice. *Nature Genet.* **43**, 1266-1269 (2011).

13 Che, R. *et al.* Control of grain size and rice yield by GL2-mediated brassinosteroid responses. *Nature plants* **2**, 15195 (2015).

14 Duan, P. *et al.* Regulation of OsGRF4 by OsmiR396 controls grain size and yield in rice. *Nature plants* **2**, 15203 (2015).

15 Wang, Y. *et al.* Copy number variation at the GL7 locus contributes to grain size diversity in rice. *Nature Genet.* **47**, 944-948, doi:10.1038/ng.3346 (2015).

16 Wang, S. *et al.* The OsSPL16-GW7 regulatory module determines grain shape and simultaneously improves rice yield and grain quality. *Nature Genet.* **47**, 949-954 (2015).

17 Zhou, Y. *et al.* Natural variations in SLG7 regulate grain shape in rice. *Genetics* **201**, 1591-1599 (2015).

18 Si, L. *et al.* OsSPL13 controls grain size in cultivated rice. *Nature Genetics* **48**, 447-456, doi:10.1038/ng.3518 (2016).

19 Kitagawa, K. *et al.* A novel kinesin 13 protein regulating rice seed length. *Plant Cell Physiol.* **51**, 1315-1329 (2010).

20 Segami, S. *et al. Small and round seed 5* gene encodes alpha-tubulin regulating seed cell elongation in rice. *Rice* **5**, 4, doi:doi:10.1186/1939-8433-5-4 (2012).

21 Duan, P. *et al.* SMALL GRAIN 1, which encodes a mitogen-activated protein kinase kinase 4, influences grain size in rice. *Abstract workform* **77**, 547-557 (2014).

22 Ishimaru, K. *et al.* Loss of function of the IAA-glucose hydrolase gene TGW6 enhances rice grain weight and increases yield. *Nature Genet.* **45**, 707-711 (2013).

23 Nakagawa, H. *et al. SHORT GRAIN1* decreases organ elongation and brassinosteroid response in rice *Plant Physiol.* **158**, 1208–1219, doi:10.1104/pp.111. 187567 (2012).

24 Li, J. *et al.* The rice HGW gene encodes a ubiquitin-associated (UBA) domain protein that regulates heading date and grain weight. *PloS one* **7**, e34231 (2012).

25 Wang, E. *et al.* Control of rice grain-filling and yield by a gene with a potential signature of domestication. *Nature Genet.* **40**, 1370-1374, doi:10.1038/ng.220 (2008).

26 She, K.-C. *et al.* A novel factor FLOURY ENDOSPERM2 is involved in regulation of rice grain size and starch quality. *The Plant Cell* **22**, 3280-3294 (2010).

27 Abe, Y. *et al.* The SMALL AND ROUND SEED1 (SRS1/DEP2) gene is involved in the regulation of seed size in rice. *Genes & Genetic Systems* **85**, 327-339, doi:10.1266/ggs.85.327 (2010).

28 Tanabe, S. *et al.* A novel cytochrome P450 is implicated in brassinosteroid biosynthesis via the characterization of a rice dwarf mutant, dwarf11, with reduced seed length. *The Plant Cell* **17**, 776-790 (2005).

29 Xu, F. *et al.* Variations in CYP78A13 coding region influence grain size and yield in rice. *Plant, cell & environment* **38**, 800-811 (2015).

30 Fang, N. *et al.* SMALL GRAIN 11 Controls Grain Size, Grain Number and Grain Yield in Rice. *Rice* **9**, 64 (2016).

31 Sun, L. *et al.* GS6, a member of the GRAS gene family, negatively regulates grain size in rice. *J. Integr. Plant Biol.* **55**, 938-949, doi:10.1111/jipb.12062 (2013).
